# Effects of topographic complexity on space-use by a key intertidal grazer in artificial environments

**DOI:** 10.1186/s40462-026-00629-x

**Published:** 2026-02-07

**Authors:** Charlotte H. Clubley, Louise B. Firth, Antony M. Knights

**Affiliations:** 1 School of Biological and Marine Sciences, University of Plymouth, Plymouth, PL4 8AA UK; 2 Department of Ecoscience, Aarhus University, Frederiksborgvej 399, Roskilde, 4000 Denmark; 3 School of Biological, Earth and Environmental Sciences, University College Cork, North Mall, Cork, Ireland

**Keywords:** Movement ecology, Habitat complexity, Spatial configuration, Correlated random walk, Limpets, Artificial structures, Eco-engineering, Habitat connectivity

## Abstract

**Background:**

The movement of animals in intertidal environments underpins community structure and is influenced by both abiotic and biotic factors within the environment. Whilst the effects of body size, tidal regime and temperature on the timing and ability of movement have been well-explored, physical features of the environment that generate complexity have received comparatively less attention. Given alterations to the topographic complexity of intertidal habitats associated with climate change and anthropogenic activities, understanding the effects of varying levels of topographic complexity on the movement of intertidal animals is critical to our understanding of biodiversity and ecosystem functioning in these habitats going forward.

**Methods:**

We experimentally manipulated topographic complexity using bespoke concrete panels at two scales, the individual panel and configuration of panels in a modelled 2 × 4 m area, to explore the effects of topographic complexity on the movement of a key intertidal gastropod and ecosystem engineer, the common limpet *Patella vulgata*. Using a combination of time-lapse photography and correlated random-walk modelling, we ‘scaled-up’ these experimental results to model the effects of topographic complexity on movement and space-use at landscape scales.

**Results:**

Results revealed a significant effect of topographic complexity on limpet movement, with greater distances travelled on ‘intermediate’ topographic complexity surfaces (surface areas = 0.08 m^2^ and 0.09 m^2^), but more frequent, shorter movements on lowest topographic complexity (surface area = 0.06 m^2^). At landscape scales, panels with intermediate topographic complexity placed in random configurations at highest spatial cover induced the greatest path lengths of simulated limpets.

**Conclusions:**

This study and its results represent the first experimental assessment of the direct effects of eco-engineered topographic complexity on the small-scale movements of intertidal gastropods and outline a first step towards the mechanistic extrapolation of results to heterogenous landscapes to explore the space-use of gastropods across larger spatial scales. With ongoing changes to intertidal environments resulting from climate change and anthropogenic activities, understanding how the movement of animals is impacted by variation in topographic complexity is essential to predicting future impacts on metapopulation dynamics and ecosystem functioning.

**Supplementary Information:**

The online version contains supplementary material available at 10.1186/s40462-026-00629-x.

## Background

Animal movement is a fundamental process underpinning metapopulation dynamics and coexistence in heterogeneous landscapes [1–4]. Animal movement primarily occurs in response to the patchy distribution of a limiting resource over a range of temporal and spatial scales [5], with motivations ranging from the search for food or mates [6], avoidance of predators [7, 8], seeking refuge from environmental stress [9], or exploration of the environment. Climate change-driven alterations to natural habitats are modifying the availability of such resources, and in many cases increasing patchiness [8, 10]. Consequently, understanding how animals respond to the distribution and amount of resources is of critical importance.

In intertidal environments, the movement of an individual may be driven by a number of biotic (e.g., competition), abiotic (e.g., tidal regime, temperature), or intrinsic (e.g., endogenous rhythms) factors, the direct and indirect effects of which are well-reported [8, 11–14]. For example, high temperatures can increase the rate of an individual’s biogeochemical reactions, allowing for greater activity [15, 16], and larger animals generally have greater energy requirements that might necessitate more frequent foraging excursions [17]. However, the effects of topographic complexity (the diversity and arrangement of three-dimensional structural features over a surface) on the movement of intertidal animals have scarcely been directly measured (but see early work from [18–20]).

Heterogeneity in intertidal substrate topography gives rise to habitat features at the mm-cm scale and manifests as crevices, pits, and pools that, in addition to offering refuge from environmental stress [21, 22], influence the space-use of mobile organisms. Rough (complex) rock surfaces, crevices, and biogenic structures (e.g., barnacles, mussels, polychaete tubes) can promote more tortuous (i.e., departure from straightness) movement paths between locations due to avoidance of these surfaces [18] or reduce the duration of periods of activity [23]. This is particularly the case for intertidal gastropods, for which suction between the muscular foot and the substrate is vital for efficient movement ([24, 25]). Movement of these gastropods is primarily associated with foraging excursions [18], and thus such barriers can cause either reduced spatial patchiness of grazing as individuals are forced to increase the complexity of their grazing excursions [18, 26], or increased patchiness as grazing becomes focused in restricted areas [27]. Consequently, small-scale topographic complexity can act as a mediator of grazing pressure and therefore dramatically alter community structure [28–30].

In addition to small-scale topographic complexity, previous studies have demonstrated that changes to the spatial configuration of topographic complexity at larger spatial scales, i.e., within a landscape (a heterogeneous area, where the type and degree of heterogeneity depends on the focal species;[31]), influences the movement patterns and abilities of individuals [2, 32–34]. For example, organisms may be reluctant to cross habitat boundaries or patches may become separated by distance [2, 35, 36]. Attempts to quantify movement over larger spatial scales have, to date, not been feasible due to the manipulation of natural habitats that would be required. Identifying a method to examine the effects of topographic complexity on animal movement that could be mechanistically ‘scaled-up’ to explore patterns over greater spatial scales is not only theoretically appealing but would have critical applications in several other fields, including landscape management, population ecology, and engineering [2, 37].

Here, we used bespoke concrete panels to manipulate and examine the effects of topographic complexity on movement and space-use of the common limpet, *Patella vulgata*. Often used as a model organism in investigations of the behaviour of intertidal gastropods (e.g., [23, 30, 38]), P. *vulgata* is a key grazer that plays an important role in habitat modification and community structuring [39–41]. Using a laboratory experiment, we aimed to characterise the effects of artificial topographic complexity (three-dimensional structural features created using artificial materials, such as concrete) on the movement of intertidal gastropods by examining the movement of *P. vulgata* on four designs of concrete panels with increasing topographic complexity (ridges and crevices). Further, we used results from the laboratory experiment to parameterise spatially explicit simulations of movement of *P. vulgata* across an experimental model grid of 2 × 4 m, with the aim to test the effects of ‘landscape-scale’ variability in the total area and arrangement of artificial topographic complexity on movement and space-use.

## Methods

### Manipulating topographic complexity

**Fig. 1:**
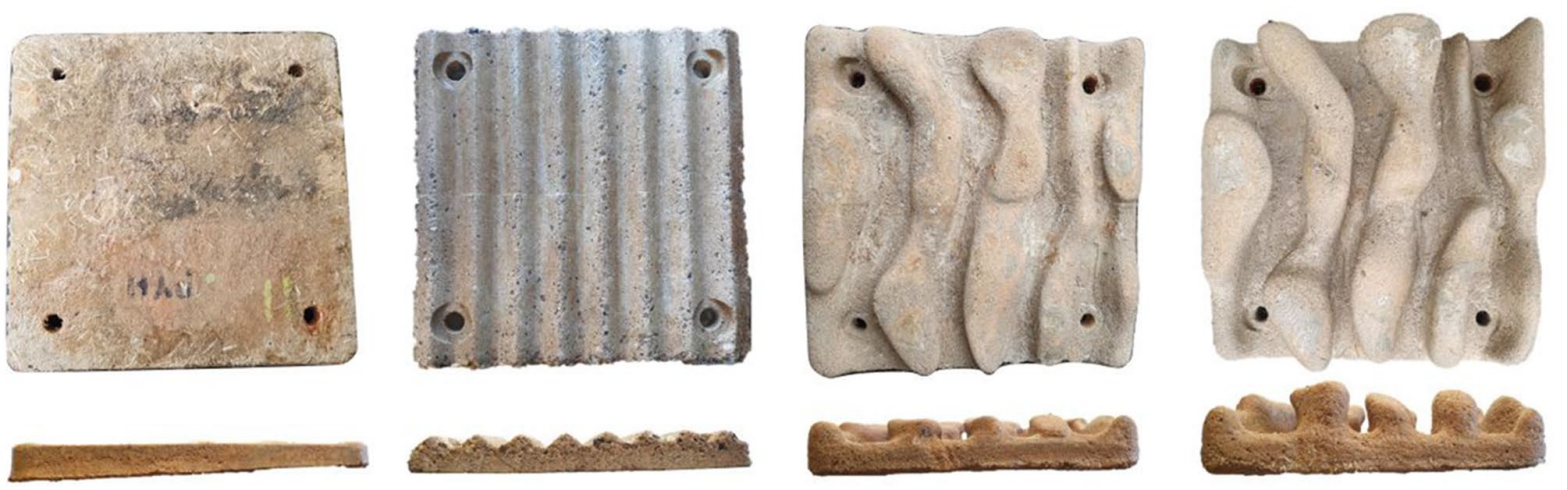
Aerial (top row) and profile (bottom row) views of the four panel types on which limpet movement was assessed. Panels from left to right: Flat, Ripple, 2.5 cm high, 5 cm high. *Photo credits: Charlotte Clubley*

To test the effects of artificial topographic complexity on limpet movement, we used four 0.25 × 0.25 m concrete panels that were: (i) flat (without crevices or ridges; surface area = 0.06 m^2^); (ii) ripple (eight 1 cm high ridges spaced 3.1 cm apart; surface area = 0.08 m^2^); (iii) 2.5 cm high (five 2.5 cm high and 1.7–6.5 cm wide ridges, separated by 1.5–5 cm wide crevices; surface area = 0.09 m^2^); or (iv) 5 cm high (five 5 cm high and 1.7–6.5 cm wide ridges, separated by 1.5–5 cm wide crevices; surface area = 0.1 m^2^) (Fig. 1). Flat, 2.5 cm high and 5 cm high panels were developed and manufactured by Reef Design Lab (Melbourne, Australia). The ripple panel was manufactured by ARC Marine LTD (Torquay, United Kingdom). All panels had a similar microtexture of < 1 mm grooves, which are unlikely to affect movement [42]. Four months prior to the experiment start (December 2022), the four panels were placed in a sump tank to allow an algal biofilm to develop to promote limpet movement in search of food.

### Live specimen collection and habituation

At the beginning of each experimental run (eight total between April and July 2023), 16 adult *P. vulgata* (length 1.2–5.0 cm) were collected during low tide from a flat, smooth, vertical seawall at Coxside, Plymouth, UK (50.3661°N, 4.1308°W) and transported on foot to the University of Plymouth’s Marine Station (~ 0.2 km from collection location; Figure S1). Only visibly unharmed individuals were retained, with damaged limpets returned to the seawall. Prior to experiments, limpets were held for a habituation period of 3 days in a tank (length × breadth × depth = 60 × 35 × 15 cm; 16 ind. tank^− 1^) located in an outdoor, sheltered laboratory, where they could move freely between submerged and exposed conditions. The tank was connected to a flow-through system in which seawater was fed directly from the rocky shore. During this time, they were starved to encourage browsing/grazing movement once experiments commenced.

### Movement assays

**Fig. 2:**
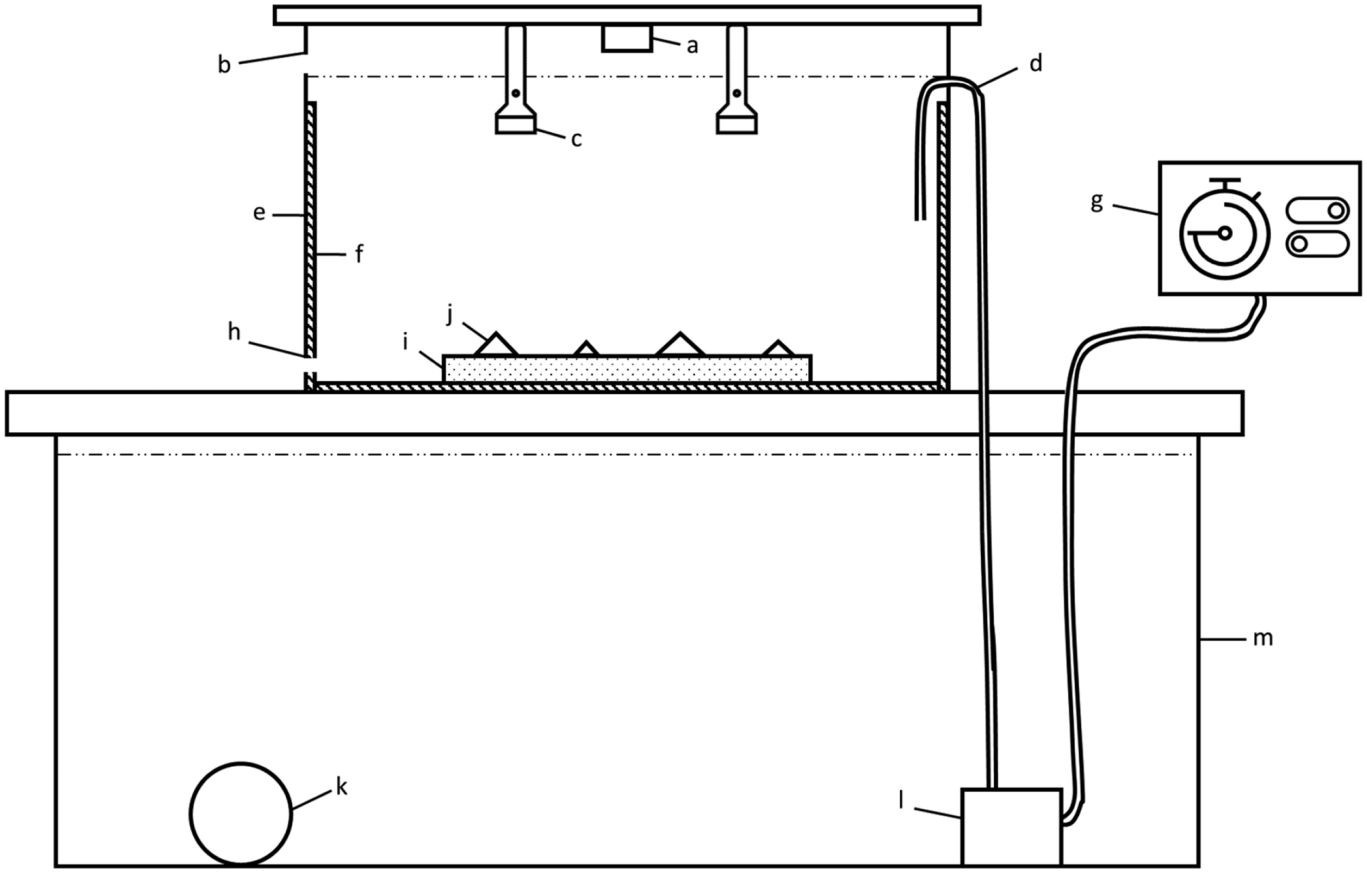
Experimental system. a = GoPro Hero 4^®^; b = overflow outlet; c = infrared flashlight; d = inlet hose; e = experimental tank; f = plastic mesh cage; g = plug-in timer; h = low tide outlet; i = complexity panel; j = limpet; k = air stone; l = Eheim pump; m = sump tank. Diagram not to scale

Experiments took place in a tidal tank system consisting of four tanks (60 × 40 × 42 cm; ~66 L tank^− 1^), one for each panel type, connected to the same flow-through seawater system as for the habituation tank (Fig. 2). We deliberately did not offer limpets a choice between complex and simple substrates, instead forcing movement within controlled topographic settings. This design choice was essential as allowing limpets to freely select their habitat could have resulted in limited or no movement, thereby obscuring how artificial topographic complexity influences movement behaviour. By constraining their options, we ensured that limpets were compelled to move, enabling us to directly measure the effects of artificial topographic complexity on movement patterns and space use. This also allowed for a mechanistic understanding of how physical habitat structure shapes animal movement which would have been difficult to obtain through a choice experiment.

A 24-hour semi-diurnal tidal regime (6-hr emersed, 6-hr immersed, twice daily) was replicated within the tanks using Eheim pumps connected to timers. Seawater from a sump tank was pumped into the tanks during periods of immersion (ca. 25 min to fill) and was drained through an outlet hole during periods of emersion (ca. 105 min to drain; Figure S2). These periods were set to mimic the timing of natural high and low tide as closely as possible. Experimental tanks were situated in a sheltered outdoor laboratory, and thus limpets experienced a natural photoperiod, and ambient temperatures mimicked those of the natural environment (8.9 –25.6 °C). A plastic mesh ‘cage’ was inserted within each tank to prevent limpets from leaving the experimental device. At no point during any of the experimental runs were limpets observed leaving the concrete panels.

One hour prior to each run, four limpets (see Figure S3 for size distribution) were placed at random positions on each panel, determined by overlaying a 5 cm × 5 cm grid on the panel and using a random number generator to select starting coordinates. Using this method, limpets on the 2.5 cm high and 5 cm high panels had an equal chance of being placed on a ridge (*n* = 16 and *n* = 15, respectively) or in a crevice (*n* = 13 and *n* = 12, respectively). A GoPro Hero 4^®^ camera retrofitted with an infrared (IR) pass lens and a Blink GoPro Camera Controller (CamDo©, https://cam-do.com/) was suspended above each experimental tank (43 cm from base of tank), alongside two IR flashlights which enabled capture of nocturnal movements. IR is unobtrusive to limpets and therefore does not affect natural behaviour [14]. Following the one-hour acclimatisation period, cameras were programmed to take a photo once every five minutes for a duration of 24 h, resulting in ~ 288 photos per panel, per run (See Figure S4 for examples). During this time, air/seawater temperature in each tank was recorded once every five minutes (in alignment with the photographs) using EnvLoggers (ElectricBlue©, https://electricblue.eu/envloggers).

For each experimental run, time-lapse photos from each panel were converted to video files using the Microsoft Windows Photo application. As immersion in seawater distorted the image ratio, the videos were separated into four periods (low tide 1, high tide 1, low tide 2, high tide 2), each containing ~ 78 images, to avoid affecting distance calculations. Across the eight runs, this produced 108 videos: 28 videos for the flat, 2.5 cm high and 5 cm high panels (seven replicates each), and 24 videos for the ripple panel (six replicates), for which IR flashlights failed during one replicate making data unusable.

Limpets were tracked in time-lapse videos using Ethovision XT (13.0) software (see Figure S5 for example). Use of Ethovision for tracking animals under a tidal regime is relatively novel (but see [14]) and thus software calibration was required to maximise performance, details of which can be found in the Supplementary Material (S1). For the four limpets in each video, Ethovision analysis resulted in an x-coordinate, y-coordinate, and the distance (cm) moved from the previous position for each frame of the video (10 FPS). Two measures of distance were calculated: gross (total) distance, calculated by summing the distance moved by the limpet between each time point of the 24-hour period; and net distance, calculated as the straight-line distance between the initial position of the limpet and its position after 24-hours.

#### Data analysis

The effects of panel type on gross and net distance moved by each limpet over the 24-hour period were analysed using linear mixed effects models (LME), estimated using restricted maximum likelihood (REML). All data were retained for analysis, including limpets that did not exhibit any movement over the 24-hour period (*n* = 7). Gross and net distances were fourth root transformed to meet assumptions of normality. Models included the factors: panel type (fixed; flat, ripple, 2.5 cm high, 5 cm high), experimental run (random; 1–8), limpet size (cm) as a continuous covariate, and month of year as a fixed covariate. Tukey’s *post-hoc* comparisons were used to investigate sources of significant effects. All models were constructed in R (Version 4.3.3, [43]) using the ‘nlme’ package [44].

As data describing the distance moved by limpets for each time step (5-min) of the 24-hour time lapse were highly zero-inflated, a binary response variable scoring 1 for movement and 0 for no movement was created. The response variable was logit transformed and the effects of panel type (as above) and immersion (fixed; immersed, emersed) modelled using an LME, also including the interaction between panel type and immersion, experimental run (random; 1–8), limpet ID (random; arbitrary; nested in experimental run), month of the year as a fixed covariate, and temperature (°C), limpet size (cm), and time of day (24-hour) as continuous covariates. Temporal autocorrelation in the data was accounted for by fitting an autoregressive moving average (ARMA) correlation structure, using AIC and likelihood ratio tests to select the best fitting model. Tukey’s *post hoc* comparisons were used to investigate sources of significant effects. Likelihood of movement on each panel type for limpets of different shell lengths during periods of immersion and emersion was calculated by back-transforming model predictions. Predictions were made over an expanded dataset representing all limpet shell lengths from the minimum to the maximum observed value (1.7–5.0 cm) at intervals of 0.1 cm. Temperature was held at an average value (15.8 °C), time of day held constant (12:00), and month of the year set as ‘May’ due to no significant effects on movement likelihood (t(29037) = -1.51, *p* = 0.13; t(29037) = -0.27, *p* = 0.79; and t(4) = 1.06, *p* = 0.35, respectively).

### Scaling up: simulating movement in response to landscape-scale variability in complexity

**Fig. 3:**
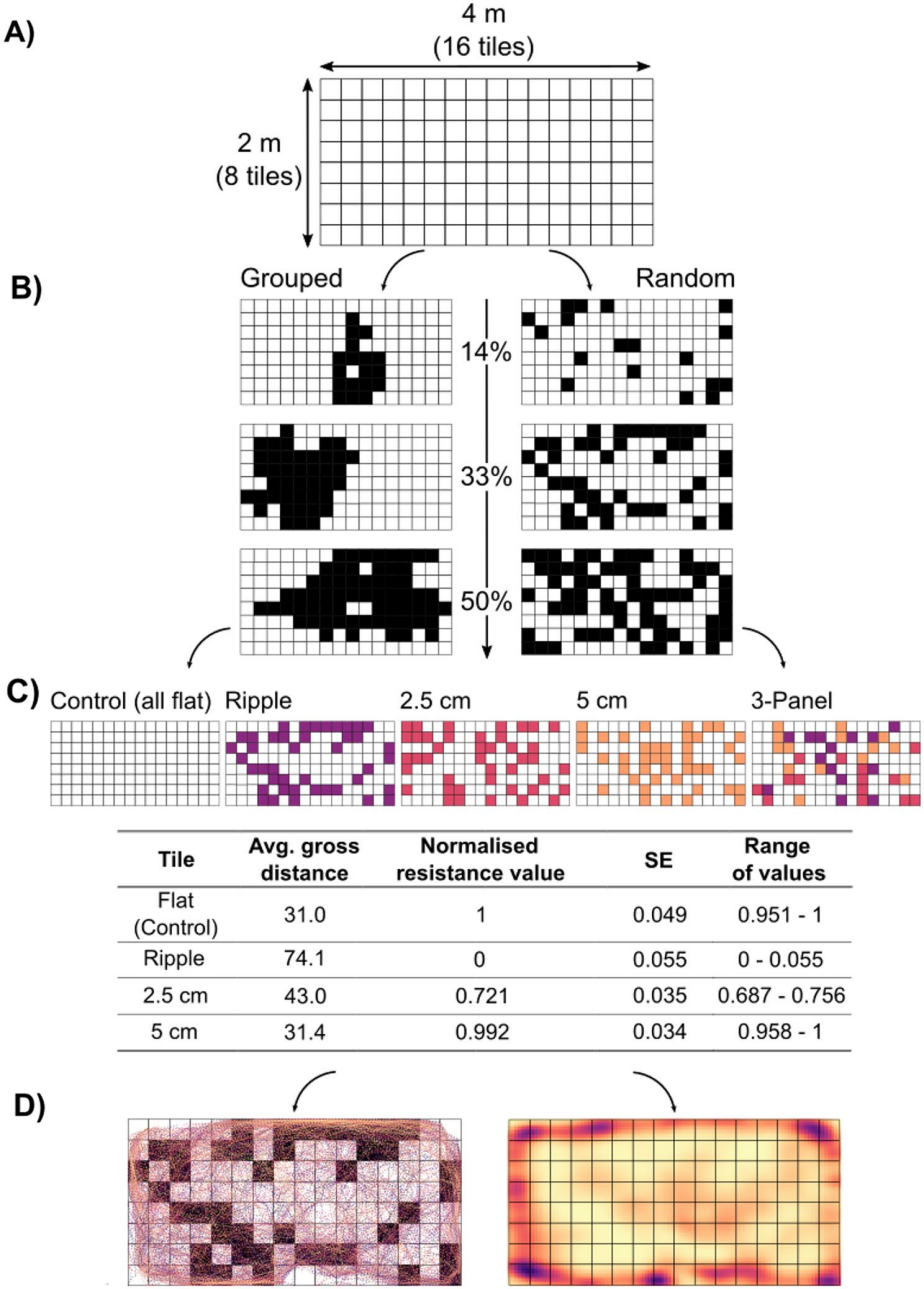
Schematic representation of the methods used to model potential limpet movement paths over an experimental model grid representing a 2 × 4 m area. (**A**) The experimental model grid on which simulations took place (8 × 16 panels representing and area of 2 × 4 m). (**B**) Examples of the spatial configuration (grouped, random) and spatial cover (~ 14%, ~ 33%, 50%) treatments that were used in simulations. Dark tiles indicate complexity panel position. (**C**) The resistance values used to quantify movement ability (physical resistance to movement) over each of the four panel types (flat, ripple, 2.5 cm high and 5 cm high). The values were informed by the gross distances travelled by limpets on each panel type in the laboratory experiment. (**D**) Examples of simulated limpet movement tracks generated using the ‘SiMRiv’ package (left), and a corresponding density map showing areas of high movement (right). In the left grid, dark tiles indicate complexity panel position

Data from the laboratory experiments were used to parameterise a spatially explicit model simulating limpet movement and space-use in response to landscape-scale variability in artificial topographic complexity. An experimental model grid of 8 × 16 panels (128 panels total representing 2 × 4 m; Fig. 3A) was generated, within which spatial cover and configuration of complexity panels was manipulated to produce six treatments (Fig. 3B). Complexity panels were either grouped (placed adjacent to one another) or randomly distributed throughout the grid at three levels of cover: ~14% (18 panels), ~ 33% (42 panels) and 50% (64 panels) (see Table S1 for associated surface areas). For each treatment a grid was created for the ripple, 2.5 cm high and 5 cm high panels, and a combination of all three panels (Fig. 3C). Flat panels, which represent the baseline topographic complexity of seawall structures, were used to represent the background landscape on which the panels were distributed, and an additional grid containing only flat panels was used as a control (Fig. 3C). This produced 25 model grids in total (6 treatments × 4 panel types + 1 control).

Potential movement and space-use of limpets over each model grid was simulated using the ‘SiMRiv’ R-package v1.0.6 ([45] Fig. 3D). To simulate movement, SiMRiv requires a ‘resistance raster’ defining the ability of an individual to move (expressed as physical resistance against movement) over different areas of the model landscape, with values constrained between 0 (no resistance, ease of movement) and 1 (high resistance, low movement). To inform these resistance values for the panels in the 25 model grids, we used the average values for gross distance moved by limpets on each of the four panel types in the laboratory experiment. We normalised the four average values (one for each panel type) and their standard error between 0 (least movement, flat panels) and 1 (greatest movement, ripple panels). Given SiMRiv’s assumption that a high resistance value (i.e., 1) indicates low movement whilst a low resistance value (i.e. 0) indicates high movement, we deemed it appropriate to invert the normalised averages by subtracting them from 1, such that the highest resistance values were assigned to the flat panel (1 ± 0.049), for which the shortest movement distances were recorded, whilst the lowest resistance values were assigned to the ripple panel (0 ± 0.055), for which the greatest movement distances were recorded (Fig. 3C). For each panel in the 25 model grids, a resistance value was assigned by randomly sampling between the lower and upper confidence intervals (± standard error) of the inverted normalised average resistance value for the focal panel type (Fig. 3C).

To simulate limpet movement, we used a correlated random walk (CRW) approach. Movement was simulated using the ‘simulate’ function of the SiMRiv package, with the behavioural state set to ‘state. CRW’ [45]. At each timestep the simulated limpets chose a direction based on an empirical probability distribution influenced by the previous heading (CRW component), the resistance value of their current panel, and the circular empirical distribution of resistance values of the surrounding panels. The correlation factor between two consecutive steps in CRW was set to 0.98, such that the paths had strong directional persistence, and the maximum step length was set to 11 cm, which was the maximum distance moved in a single timestep from the laboratory experiments. The number of steps was set to 288, matching the number of time-steps in the laboratory-based time-lapse recordings.

Based on the average density of *P. vulgata* in a preliminary survey of 10 replicate quadrats (0.5 × 0.5 m; February 2023; 8 ± 2 ind. per 0.25 m^2^), we chose to simulate the movement of 1000 limpets per treatment. Limpet starting positions were equally distributed at set intervals within the grid. At the end of each simulation, total path length (cm) of each limpet was calculated. The effects of panel type (fixed; control, ripple, 2.5 cm high, 5 cm high, 3-panel), configuration (fixed; grouped, random), cover (fixed; ~14%, ~ 33%, 50%), and the interaction between the three on path lengths of simulated limpets were analysed using a general linear model (glm) with a log-link function and gamma distribution. Tukey’s *post hoc* comparisons were used to investigate sources of significant effects.

## Results

### The effects of complexity on limpet movement

**Fig. 4:**
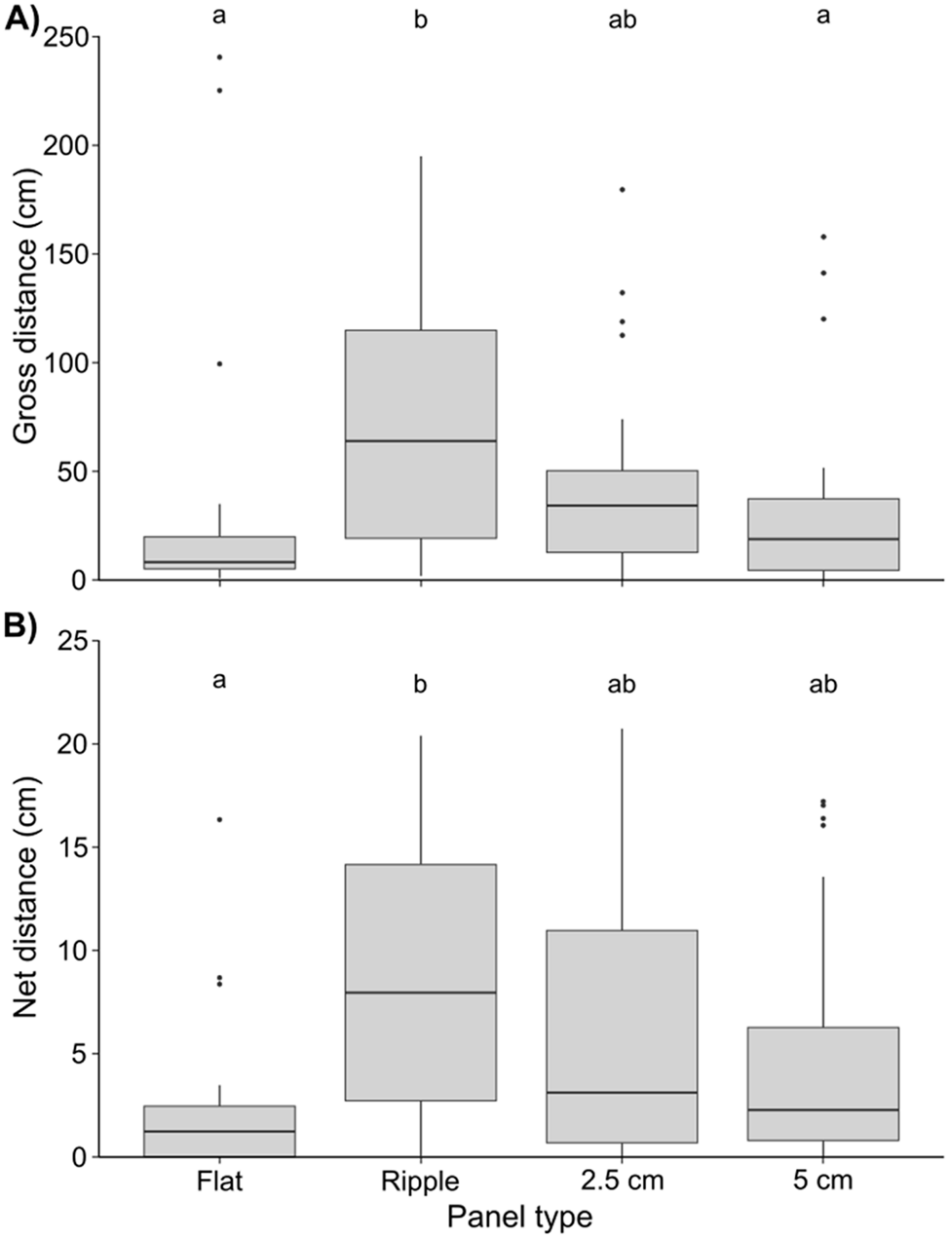
The effect of panel type on limpet movement. **(A)** The effect of panel type on gross distance (cm) moved by limpets over a 24-hour period. **(B)** The effect of panel type on net distance (cm) moved by limpets over a 24-hour period. Letters indicate significant differences between groups identified by Tukey’s *post hoc* tests

Across the eight experimental runs, we obtained and analysed movement tracks for 103 limpets. Gross distances moved by limpets varied significantly across the four panel types (t(91) = -2.41, *p* < 0.05), with limpets on the ripple panel travelling the greatest distances (median = 64.0 cm) and on average 58% further than limpets on flat or 5 cm high panels (Fig. 4a; Table S2). Whilst there was no influence of month of the year on this effect (t(4) = 0.41, *p* > 0.05), effects were dependent on limpet size, with larger limpets on all panel types travelling greater gross distances (t(91) = 4.70, *p* < 0.001) – this was most evident for the ripple, 2.5 cm high and 5 cm high panels (Figure S6). Conversely, both month of the year and limpet size had no effect on net distances (t(4) = 1.02, *p* > 0.05 and t(91) = 1.07, *p* > 0.05, respectively). Average net distance was 76% greater for ripple than flat panels (t(92) = -3.94, *p* < 0.005), but there was no difference between net distances of limpets on the ripple, 2.5 cm high and 5 cm high panels (Fig. 4b).

**Fig. 5:**
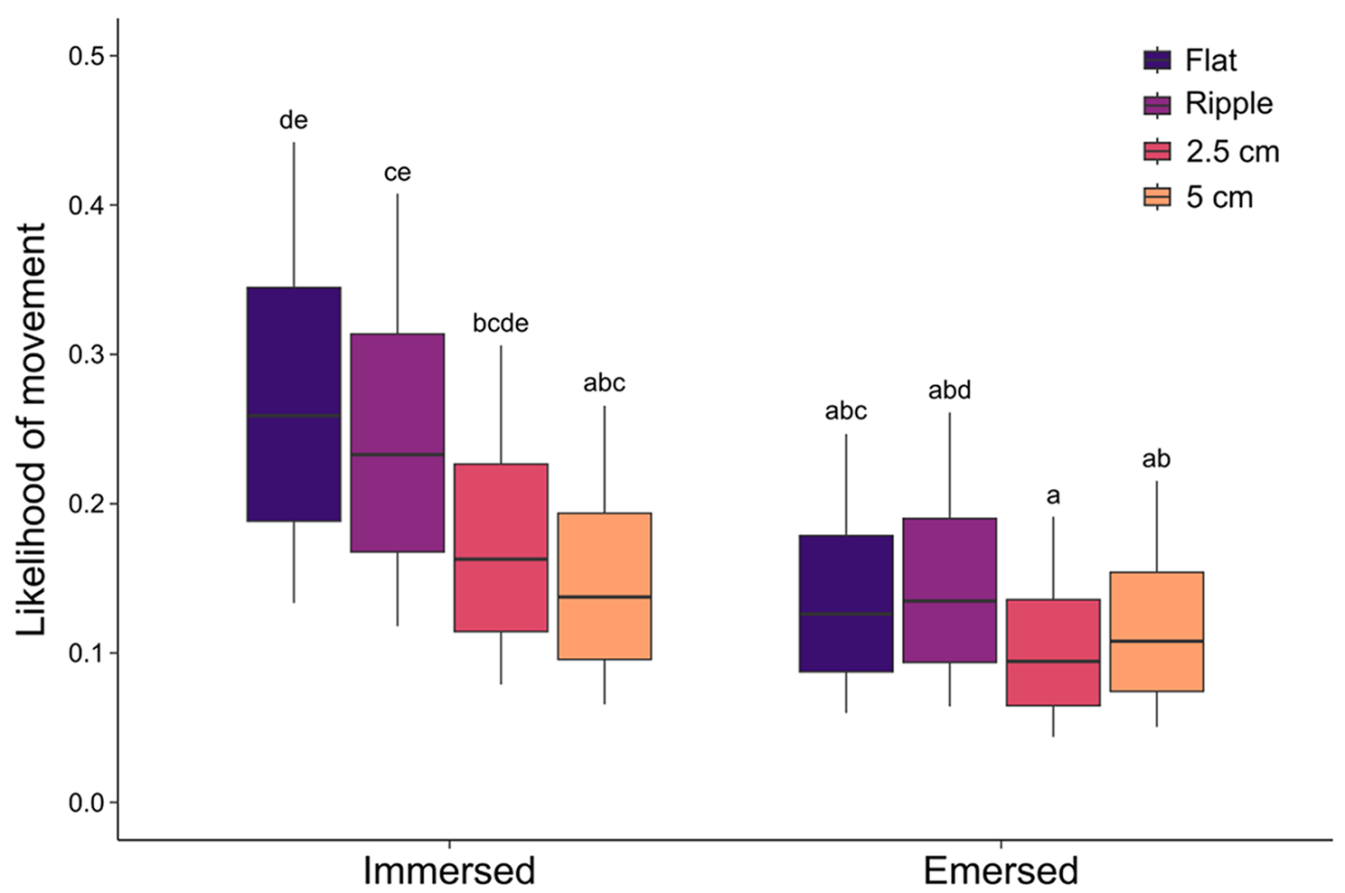
The predicted likelihood of movement of limpets on the four panels (flat, ripple, 2.5 cm high, 5 cm high) during periods of immersion and emersion. Likelihood is shown for limpets of sizes ranging from 1.7–5 cm (0.1 cm intervals) to represent the size range of limpets used in the experiment. Letters indicate significant differences between groups, identified by Tukey’s *post hoc* tests

Across all panels and experimental runs, limpet movement predominantly occurred during daytime emersion and nocturnal immersion, with very little movement during daytime immersion (Figure S7 A-D). Across all panels, the predicted likelihood of limpet movement ranged from 0.04 to 0.45. For every 0.5 cm increase in limpet shell size, there was a 1 unit increase in the likelihood of movement at each time step (t(91(= 4.61, *p* < 0.001; Figure S8), with movement significantly more likely during periods of immersion than emersion (t(91) = 4.82, *p* < 0.001), and most likely to occur on flat panels during immersion (Fig. 5; Table S3).

### Scaling up: simulating movement in response to landscape-scale variability in complexity

**Fig. 6:**
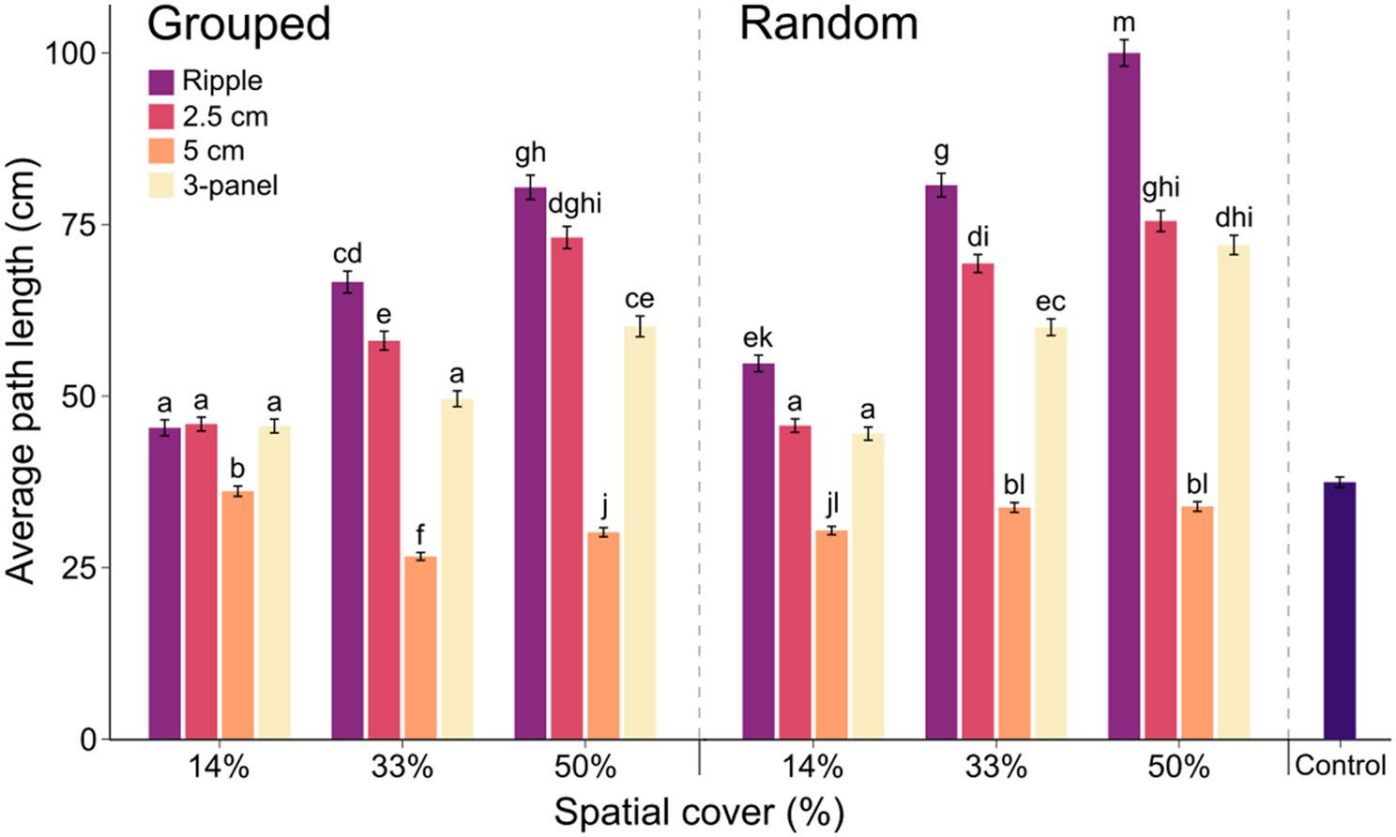
Average path length (cm) of 1000 simulated limpets over a 2 × 4 m experimental model grid containing either ripple panels, 2.5 cm high panels, 5 cm high panels, or a combination of equal numbers of all three (3-panel), placed in either grouped or random configurations at increasing spatial covers (%). Also shown is the average path length of simulated limpets in a control treatment where no eco-engineered panels were incorporated. Letters indicate significant differences between groups, identified by Tukey’s *post hoc* tests

Excluding the 5 cm high panel in grouped configurations at 14% cover, movement paths of all simulated limpets over experimental model grids significantly differed from the control treatment using only flat panels (Table S4). However, paths for grids using the 5 cm high panel were, on average 46% shorter than simulations using any of the other panel types in either configuration for all spatial covers (Fig. 6). For the ripple, 2.5 cm high and 3-panel treatments, path lengths of simulated limpets were significantly longer for panels in random than grouped configurations at 33% and 50% spatial cover (excluding the 2.5 cm high panel at 50% cover, for which path lengths were equal; t_23976_ = 6.12, *p* < 0.001; Fig. 6). Equally, across the ripple, 2.5 cm high and 3-panel treatments an increase in spatial cover from 14% to 33% to 50% generally corresponded with a significant increase in path length of simulated limpets, with the exceptions being the increases from 33% to 50% for the 2.5 cm high panel treatment in random configurations and the increase from 14% to 33% for the 3-panel treatment in grouped configurations (Fig. 6; Table S4).

For grouped configurations, there was no significant difference in path lengths between the ripple, 2.5 cm high and 3-panel treatments at 14% cover, however at 33% and 50% cover path lengths were significantly greater when models used either the ripple or 2.5 cm high panels compared to the 3-panel treatments (for 33% cover: ripple > 2.5 cm high > 3-panel; for 50% cover ripple = 2.5 cm high > 3-panel; Table S4). On the other hand, for random configurations the ripple panel produced significantly longer paths at 14% cover, which remained the case at 33% (ripple > 2.5 cm high > 3-panel) and 50% cover (ripple > 2.5 cm high = 3-panel; Table S4). For models using the ripple panel, path lengths were significantly greater for random configurations at all spatial covers (Fig. 6). For the 2.5 cm high panel, there was no significant difference at 14% and 50% cover, but at 33% cover random configurations produced significantly longer paths. Finally, for the 3-panel treatment, paths were significantly longer for random configurations at 33% and 50% cover (Fig. 6).

**Fig. 7:**
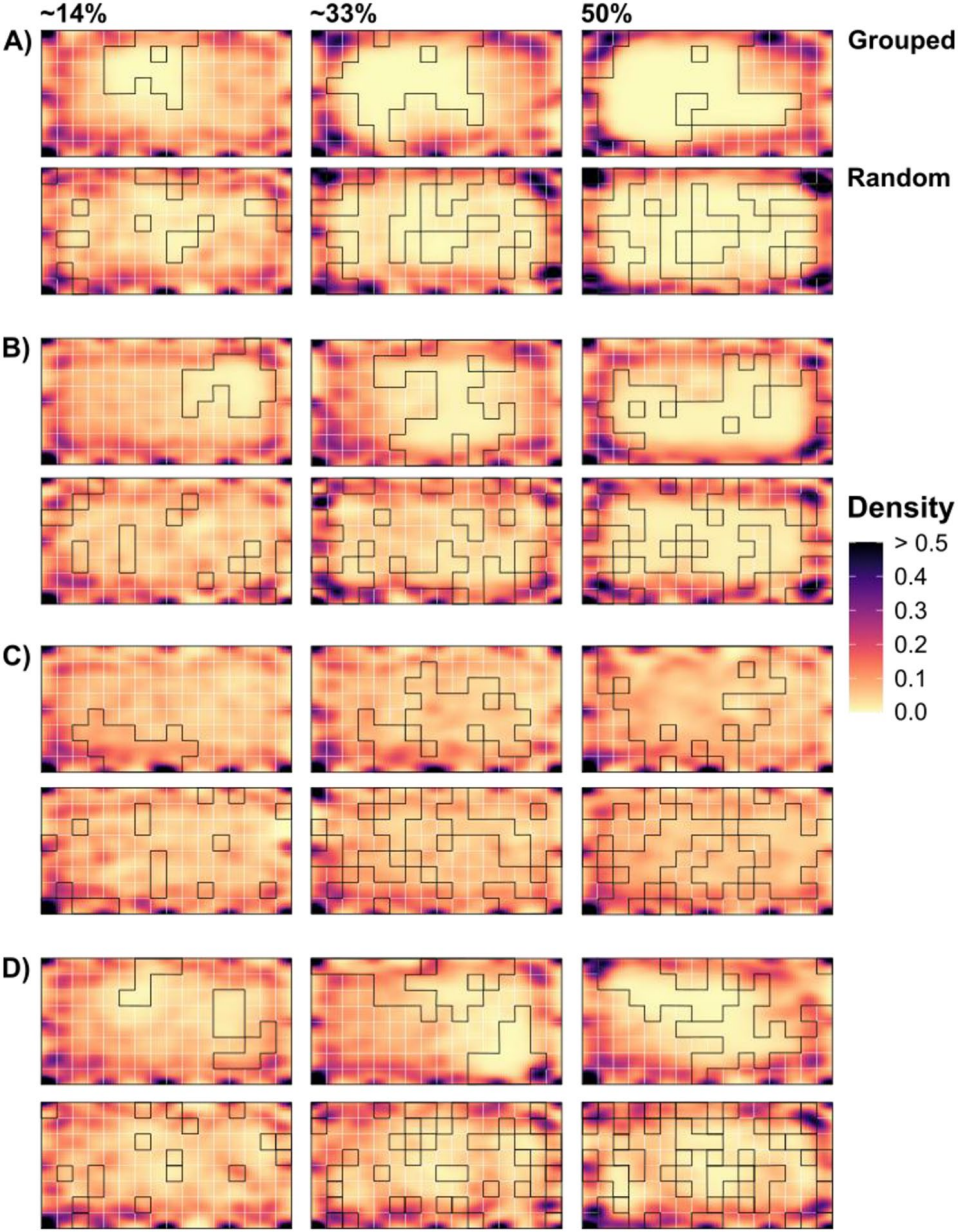
Density maps showing the distribution of 1000 simulated paths of limpets on a 2 × 4 m experimental model grid. Black lines outline the placement of complexity panels within the grid. (**A**) Density maps of simulated paths for grids containing ripple panels. (**B**) Density maps of simulated paths for grids containing 2.5 cm high panels. (**C**) Density maps of simulated paths for grids containing 5 cm high panels. (**D**) Density maps of simulated paths for grids containing the 3-panel combination

When visually comparing paths, whilst an increase spatial cover and the arrangement of panels in a random configuration generally produces greater path lengths, the paths are constrained to smaller areas within the model grid (Fig. 7). Conversely, when panel cover is lower and grouped, paths appear somewhat more evenly distributed. This is illustrated by the models with the longest (ripple, random, 50%) and shortest paths (5 cm high, grouped, 33%; Fig. 7A and C, respectively). Paths of simulated limpets for model grids containing the 5 cm high panel, whilst significantly shorter than for any other panel treatment (Fig. 6), appeared to be evenly distributed across the grid (Fig. 7), likely due to similarity in the normalised resistance values (physical resistance against movement) for 5 cm high panels (0.992 ± 0.034) and the flat panels representing the background landscape of the model grid (1 ± 0.049). Conversely, simulated limpets in the other treatments tend towards avoiding areas in which complexity panels were placed despite their lower resistance values than the ‘background’ flat panels (Fig. 7). This is likely a property of the way that the SiMRiv package computes step lengths – step length is calculated as a fraction of the maximum step length (here, 11 cm) proportional to the resistance at the start and end points of the limpet. In this way, movement is likely to occur more rapidly through areas of low resistance, leading to lesser time spent in these areas compared to higher resistance areas.

## Discussion

### The effect of topographic complexity on limpet movement

In intertidal environments, physical features of the substrate such as rough rock areas and biological cover (e.g., mussels, barnacles, polychaetes) create barriers to the movement of grazing species [18, 23]. Field studies by Underwood and Chapman [19]), [20]); and Chapman [46] demonstrated that non-homing (i.e., do not return to a particular location) intertidal gastropods move greater distances on flat, smooth surfaces. In contrast, here we observed significantly shorter distances travelled by the homing limpet *P. vulgata* on flat panels, absent of topographic complexity, than those on more topographically complex panels for which ridges and crevices were present.

As an intertidal gastropod, *P. vulgata* relies on a secure attachment to the substrate for safe and efficient movement [39, 47], defence against predation (as has been shown for *Siphonaria* spp.; [48, 49]), protection from desiccation at low tide [50] and preventing dislodgement [51]. Flat, smooth surfaces, such as those often found on artificial structures in intertidal environments and represented by the flat panel in this study, generally promote greater abundances of limpets due to the ease of attachment to these substrates [39, 52]. It may be that the flat panels provided a secure attachment substrate for limpets leading to a ‘hunker down’ response to transplantation [53], whilst more topographically complex panels prompted longer exploratory movements in search of a secure attachment area [8].

Such effects have been demonstrated in natural mussel beds, where complex topography prevents the secure attachment of limpets to the substrate and yet movement persists, particularly in the case of larger limpets [54]. In the present study, the dimensions of the ridges and crevices on the ripple and 2.5 cm high panels (see Sect. 2.1 for details) may have been too small to enable larger limpets (> 2.5 cm length, representing 80% of limpets in this experiment) to attach securely. Shell size is an important determinant of spatial segregation of gastropods [55]. For example, Gray and Hodgson [56] observed a size gradient of the Patellid limpet *Helcion pectunculus* within natural crevices, with smaller individuals occupying the narrower back regions and moving towards the wider entrance as they grow. The crevices on the ripple and 2.5 cm high panels may have imposed a similar pattern of spatial segregation in our study, limiting suitable attachment space for larger limpets. Byers [57] also found a size-dependent relationship between movement of salt marsh snail species and resource levels, with larger individuals exhibiting increased dispersal at low resource levels, but no such relationship observed in smaller individuals. Thus, in our study the significant increase in gross distance with limpet shell size, which was particularly evident for the ripple and 2.5 cm high panels (Figure S6), is likely driven by size-based spatial segregation of limpets that reduces available attachment space and forces exploratory movement in search of a secure attachment location.

For the 5 cm high panel, the larger dimensions are theoretically able to accommodate the largest individuals used in this study (shell length = 5 cm), and thus despite the panel being more topographically complex than the ripple and 2.5 cm high panels it may have provided a more suitable environment for attachment, leading to the shorter observed movements similar to those on the flat panel. Alternatively, evidence from terrestrial gastropod populations suggests larger individuals, when moving over vertical surfaces, experience increased stress on their muscular foot due to the combined pressures of their own shell weight and gravity [58, 59]. Therefore, it is possible the reduced movement of limpets on the 5 cm high panel represents a strategy to avoid such stress. Evidence from Littorinid populations in natural environments suggests that shell height limits exploitation of microhabitats, with taller shells excluding individuals from narrow shelters [55]. Whilst shell height was not measured in this study, the narrow crevices on the 2.5 cm high and 5 cm high panels (width: 1.5–5 cm) may equally have influenced attachment ability, contributing to the observed differences in movement distance and warranting further investigation in future research.

Although movement distances were shortest on the flat panel, movement occurred more regularly than on the ripple or 2.5 cm high panels. A similar result was found by Santini et al. [23] in their study of *P. vulgata* on a vertical seawall, where limpets moved more readily on flat surfaces than on rough, barnacle-covered surfaces. They attributed this pattern to reduced adherence of the limpet shell to topographically complex barnacle-covered substrate. In the same study, Santini et al. [23] noted that limpets on flat surfaces moved to forage over barnacle-covered areas, but the reverse was not true. The more topographically complex substrate and greater surface area created by barnacles has been shown to support greater microalgal resources than topographically homogeneous areas [23, 60]. Whilst we allowed an algal biofilm to form on panels used in this study, we took no measurements of algal supply and hence the more topographically complex ripple, 2.5 cm high and 5 cm high panels may have provided a greater food source than the topographically simpler flat panel, prompting more exploratory movements over larger distances. However, whilst the movement of intertidal gastropods is largely associated with foraging behaviour [18, 61], we did not measure for a direct link between movement and grazing (e.g., arcuate oscillatory motion typical of limpets conducting grazing excursions; [61]) in the present study. Consequently, motivation for movement may have been driven by any number of factors linked to the artificial topographic complexity of the panels, including exploration of the environment [5, 8], or seeking shelter from perceived competition and predation [1, 7].

### Experimental considerations and future direction

A key feature of our experimental design was the deliberate omission of a choice experiment, compelling limpets to move within defined artificial topographic complexity treatments rather than allowing them to select “preferred” substrates. While this approach may have introduced a forced movement scenario [2, 62], it provided critical insights that may have been obscured if limpets were allowed to avoid movement altogether. In natural settings, limpets often remain inactive when conditions are favourable, minimising energy expenditure and exposure to predation (e.g., by “hunkering down” on secure substrates) [53, 63]. By removing the option to remain inactive or select optimal microhabitats, our design likely induced movement that can be interpreted either as an exploratory response to unfamiliar or suboptimal substrate, or as a stress response aimed at locating suitable attachment sites. This forced movement approach thus revealed behavioural adjustments to artificial topographic complexity that would otherwise be masked by inactivity or habitat choice.

Indeed, the greater distances moved on ‘intermediate’ artificial topographic complexity panels (i.e., the ripple (surface area = 0.08 m^2^) and 2.5 cm high (surface area = 0.09 m^2^) panels) may reflect increased locomotory effort associated with finding secure attachment points, highlighting the costs of complex topography. Conversely, reduced movement on flat or highly topographically complex panels may indicate more secure attachment or “settling” behaviours, suggesting a nuanced relationship between movement and substrate quality that would be difficult to resolve without this forced movement framework. Although we did not directly measure motivation (e.g., foraging vs. stress-related movement), our approach provides a foundational mechanistic understanding of how artificial topographic complexity shapes limpet movement patterns.

Future work could build upon this by incorporating choice experiments to complement forced movement data, potentially disentangling the relative influences of habitat preference, stress, and foraging motivation. Measuring grazing rates across different surface topographies would add critical information. Additionally, exploring how movement patterns change with acclimation or over longer timescales may reveal whether initial forced movement transitions into more selective or efficient behaviours [47, 64]. Such longer-term studies would also contribute to identifying seasonal variability in the response of movement to artificial topographic complexity, as past studies have demonstrated significant variation in activity of *P. vulgata* between spring, summer and autumn periods [13, 17]. Finally, in natural settings the inclination of rock surfaces has been shown to affect timing and patterns of limpet activity [13]. Although methodological constraints in the present study necessitated limpets were placed horizontally despite being collected from vertical surfaces, future work examining movement at different inclinations would determine whether this effect interacts with artificial topographic complexity. Together, such studies would deepen our understanding of the ecological and evolutionary consequences of habitat complexity in shaping the movement ecology of intertidal species.

### Scaling up: potential space-use in a model landscape

The pattern, benefits and risks of animal movement depend on landscape structure, including the quality of habitat, total habitat area, and the spatial configuration of habitat patches within a landscape [32]. Previous studies of the effects of total habitat area on biodiversity in natural environments have found them to be intensified by the configuration of habitat patches within the larger landscape [65–68]. Similarly, in this study effects of both configuration and the artificial topographic complexity of panels on path length of simulated limpets were dependent on the spatial cover of the panels. It is believed that increased isolation of habitat patches resulting from random or fragmented configurations and small habitat area produce greater movement distances as animals must travel further to reach ‘good quality’ habitat [32, 33]. Whilst in this study the paths of simulated limpets were generally shorter where panels were placed in grouped configurations, greatest path lengths of simulated limpets were for panels placed in random configurations at the greatest spatial cover (50%). The difficulty herein lies in what is considered ‘good quality’ habitat for the movement of *P. vulgata*.

Should the motivation for greater movement distances of limpets in the laboratory experiment on the ripple and 2.5 cm high panels relative to the flat and 5 cm high panels be exploration in search of a secure attachment location, one might consider flat and 5 cm high panels to represent ‘good quality’ habitat. The greater areas of flat substrate on these panels likely provides greater opportunity for limpets to form a secure attachment, as is evidenced by their dominance of artificial structures consisting of flat, smooth surfaces in the United Kingdom [39]. Similarly, Bauer et al. [69] found that limpets were more likely to be found (and occupy homescars) on patches of flatter rock, thus suggesting a ‘preference’ for flat compared to rugose substrata. In this scenario, the spatial cover of ‘good quality’ habitat (flat panels) in the experimental model grids decreases as the area of the other, more topographically complex, panel types increases. Longer paths of simulated limpets for artificial topographic complexity panels at greater spatial covers in random configurations may therefore be more costly to the individual limpet and represent ‘non-optimal movement’ [7, 32]. Further, increased spatial cover of artificial topographic complexity panels could create ‘trapping effects’, forcing limpets to remain in areas where movement is more ‘costly’ [18]. Whilst in this study we simulated movement of limpets with no defined body length, given the significance of body length to gross distance moved in the laboratory experiments and the potential implications for limpet attachment discussed in the previous section, further simulations incorporating different size categories may help to elucidate the patterns observed here.

Of course, in order to draw such conclusions and understand patterns in animal movement over landscape-scales we must first understand *why* the animals are moving [5, 70]. Here, we were unable to resolve the motivation for movement of limpets in the laboratory experiments, and thus the biological relevance of movement in our simulations remains obscured. However, this study represents a first attempt towards the extrapolation of the impacts of artificial topographic complexity on movement to landscape scales and, given refinement and further study, such methods will be critical to our ability to understand the effects of spatial variability in resources, becoming more prevalent as a result of anthropogenic activity and climate change [10], on movement and space-use of animals.

### Implications for ecological (eco-) engineering

The effects of the concrete panels we used in this study as eco-engineering interventions designed to increase native biodiversity on artificial structures such as seawalls and marinas have been well-reported in a number of spatial contexts (e.g., [71–74]). However, to date these studies have largely focused on descriptions of patterns, and rarely are explorations into the underlying mechanisms of biological community structure, such as the distribution and movement of ecosystem engineers [69], and how they might be leveraged in future eco-engineering interventions conducted. Given limpets’ role as ecosystem engineers with the ability to form “limpet barrens” (*sensu* [39]) on artificial structures and to compromise the long-term structural resilience of the structures themselves through biomechanical erosion [37], the results we present here have large potential implications for future eco-engineering design. By strategically deploying eco-engineering in such a way as to restrict limpet movement through creation of ‘non-optimal’ artificial landscapes (here, represented by complexity panels placed in random configurations at high spatial cover), it may be possible to mediate these grazing and erosive pressures. Such strategies could have knock on biodiversity benefits [39, 75] consistent with the overarching goals of eco-engineering [52, 76]. Therefore, to enhance desirable biological outcomes future eco-engineering research should consider effects of artificial topographic complexity on animal movement in both intervention design and deployment.

## Conclusion

Assessing animal movement and the factors impacting it is essential to our understanding of the processes underlying metapopulation dynamics, coexistence and biological invasions [2, 3, 8, 77]. Here, we have demonstrated that artificial topographic complexity drives differences in the movement and space-use of the common limpet, *Patella vulgata*, most likely as a result of size-based spatial segregation caused by an inability of large individuals to form secure attachments with more topographically complex substrates. This represents the first experimental assessment of the direct effects of artificial topographic complexity, produced by concrete panels, on the small-scale movements of intertidal gastropods. Additionally, the methods used here make steps towards the mechanistic extrapolation of such effects to heterogenous landscapes, exploring the potential space-use of gastropods across larger spatial scales. These findings have the potential to be extended to other gastropods and, given the importance of movement in structuring biodiversity [28, 30, 40], are a key first step towards predicting how species will respond to future changes in the spatial distribution of resources.

## Supplementary Information

Below is the link to the electronic supplementary material.

Supplementary Material 1

## Data Availability

The datasets supporting the conclusions of this article are available in the Mendeley repository, 10.17632/vdjjkfnnsp.1. The code used to carry out analysis and run movement simulations are available in the GitHub repository, https://github.com/cclubley/Clubley-et-al-Navigating-novel-habitats.
