# Supplementary material for: Effects of topographic complexity on space-use by a key intertidal grazer in artificial environments
Source: Mov Ecol. 2026 Feb 7;14:12. doi: 10.1186/s40462-026-00629-x (PMC12937512; doi:10.1186/s40462-026-00629-x)
Supplement: Supplementary file 1 — Supplementary Material 1 [file 40462_2026_629_MOESM1_ESM.docx]

**SUPPLEMENTARY MATERIAL:**

Effects of Topographic Complexity on Space-Use by a Key Intertidal Grazer in Artificial Environments

**AUTHORS**

*Charlotte H. Clubley, Louise B. Firth, Antony M. Knights*

**CORRESPONDING AUTHOR**

Dr Charlotte H. Clubley, [cclubley@ecos.au.dk](mailto:cclubley@ecos.au.dk)

Aarhus University, Department of Ecoscience, Frederiksborgvej 399, 4000 Roskilde, Denmark


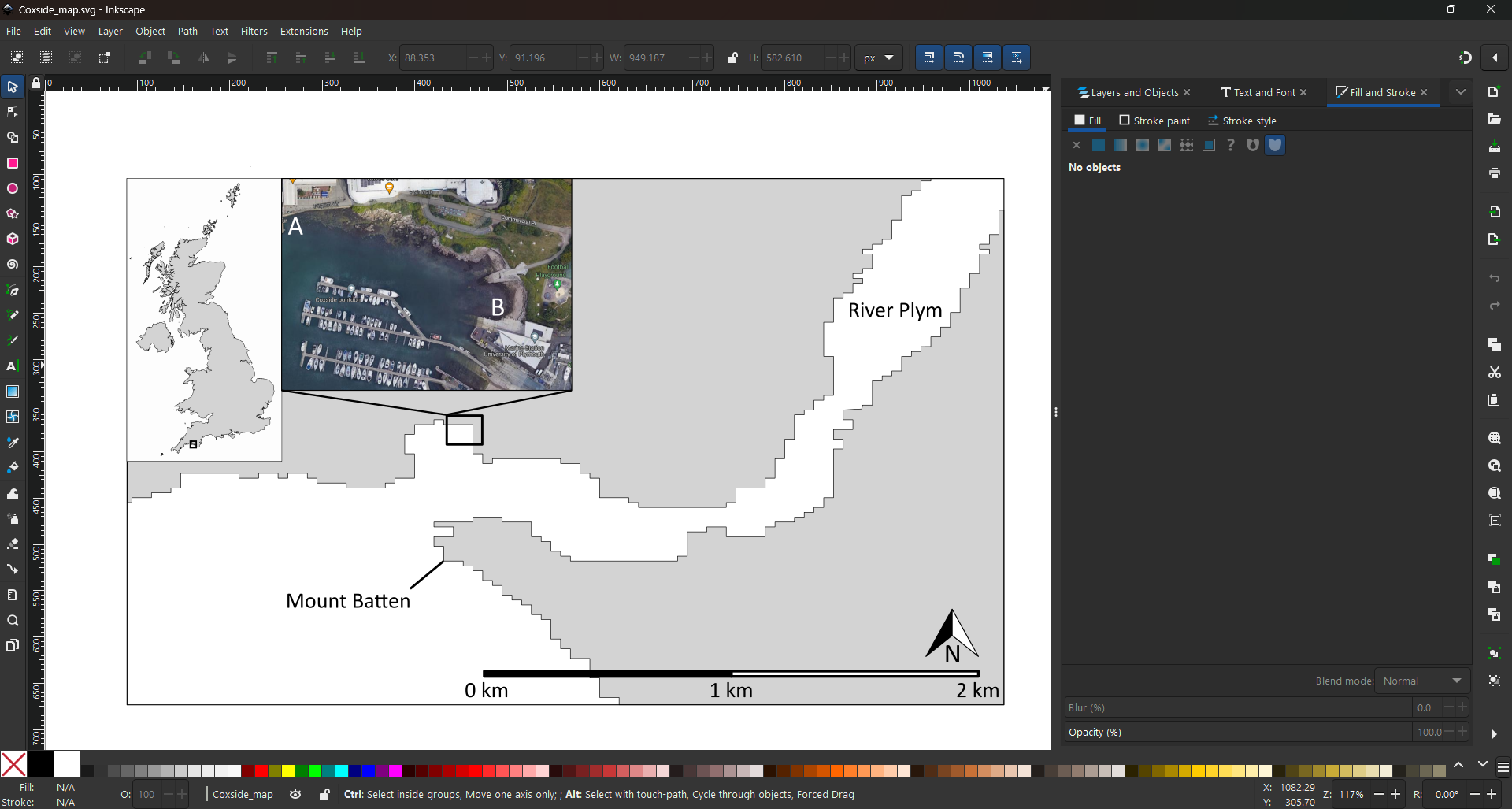


**Figure S1.** The location from which adult *Patella vulgata* used in the laboratory experiment were collected: Coxside, Plymouth, Devon, United Kingdom (50.3661°N, 4.1308°W). Insets show the location of Plymouth in the United Kingdom and a map of Coxside (© Google Maps). A = the location of the seawall from which limpets were collected. B = the location of the University of Plymouth’s Marine Station.


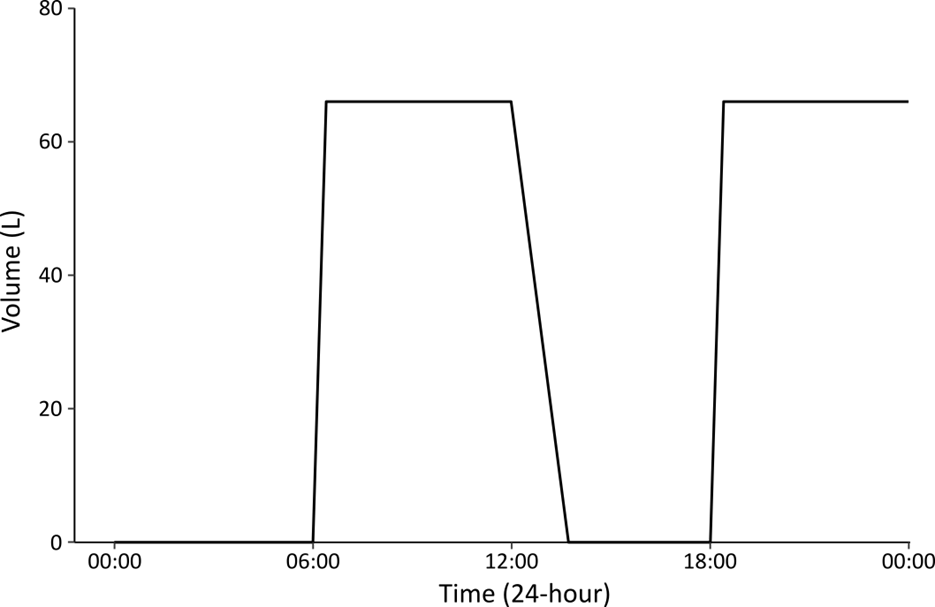


**Figure S2.** An example of the semi-diurnal ‘tidal regime’ in experimental tanks over a period of 24-hours.


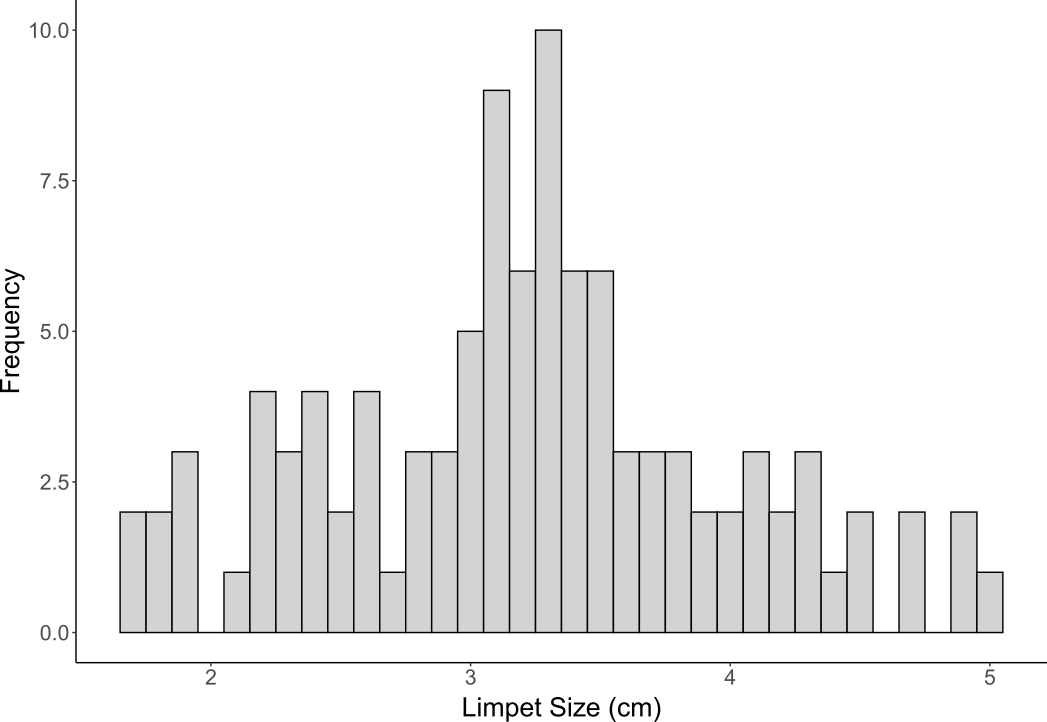


**Figure S3.** Frequency distribution of body size (width, cm) of limpets used in the laboratory experiments. Body size ranged from 1.3 – 5.0 cm.


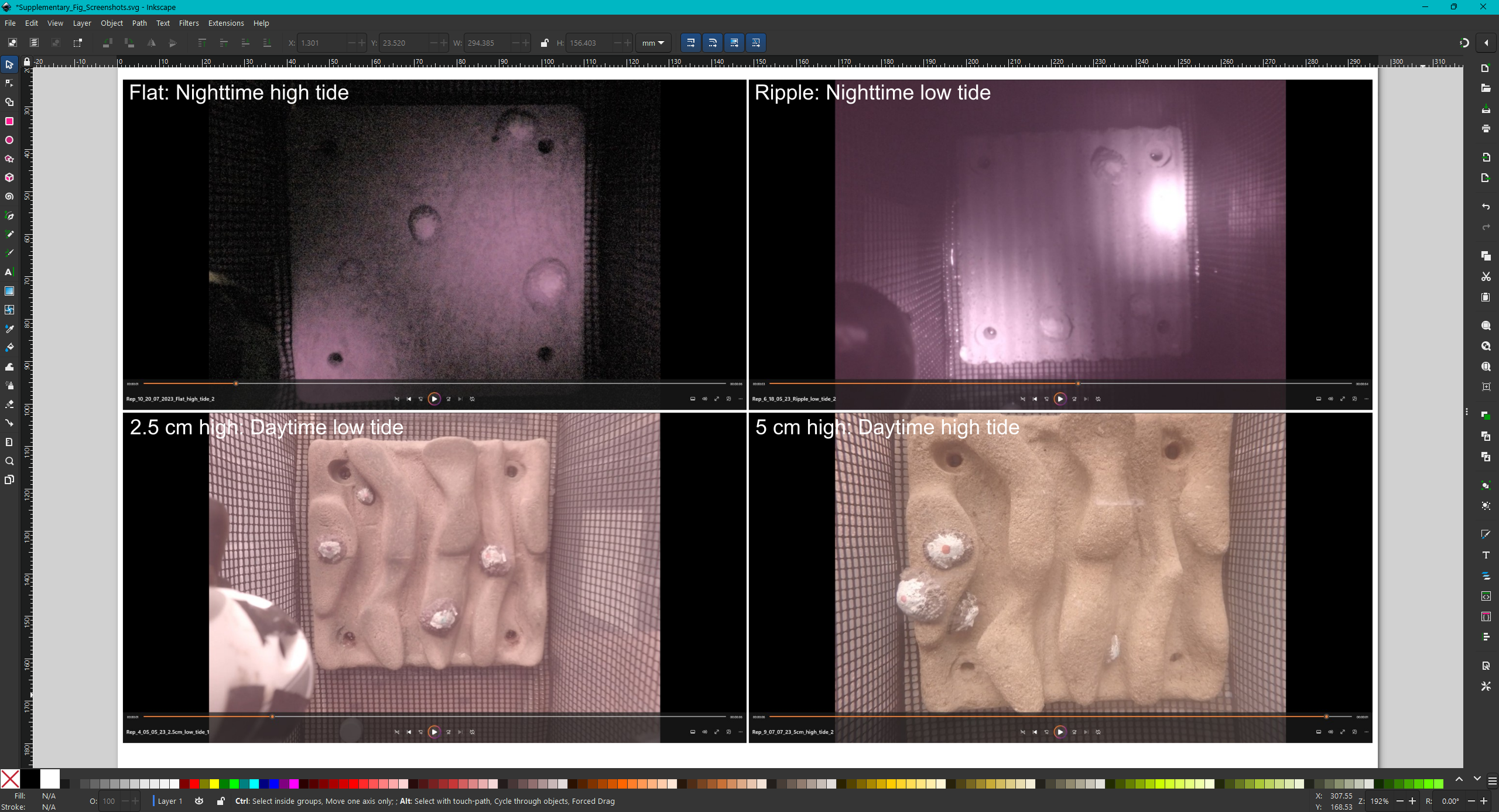


**Figure S4.** Example photos from the laboratory timelapse recordings of limpet movement. An example is shown for each panel type (flat, ripple, 2.5 cm high, 5 cm high) from each distinct period (nighttime high tide, nighttime low tide, daytime low tide, daytime high tide).

*
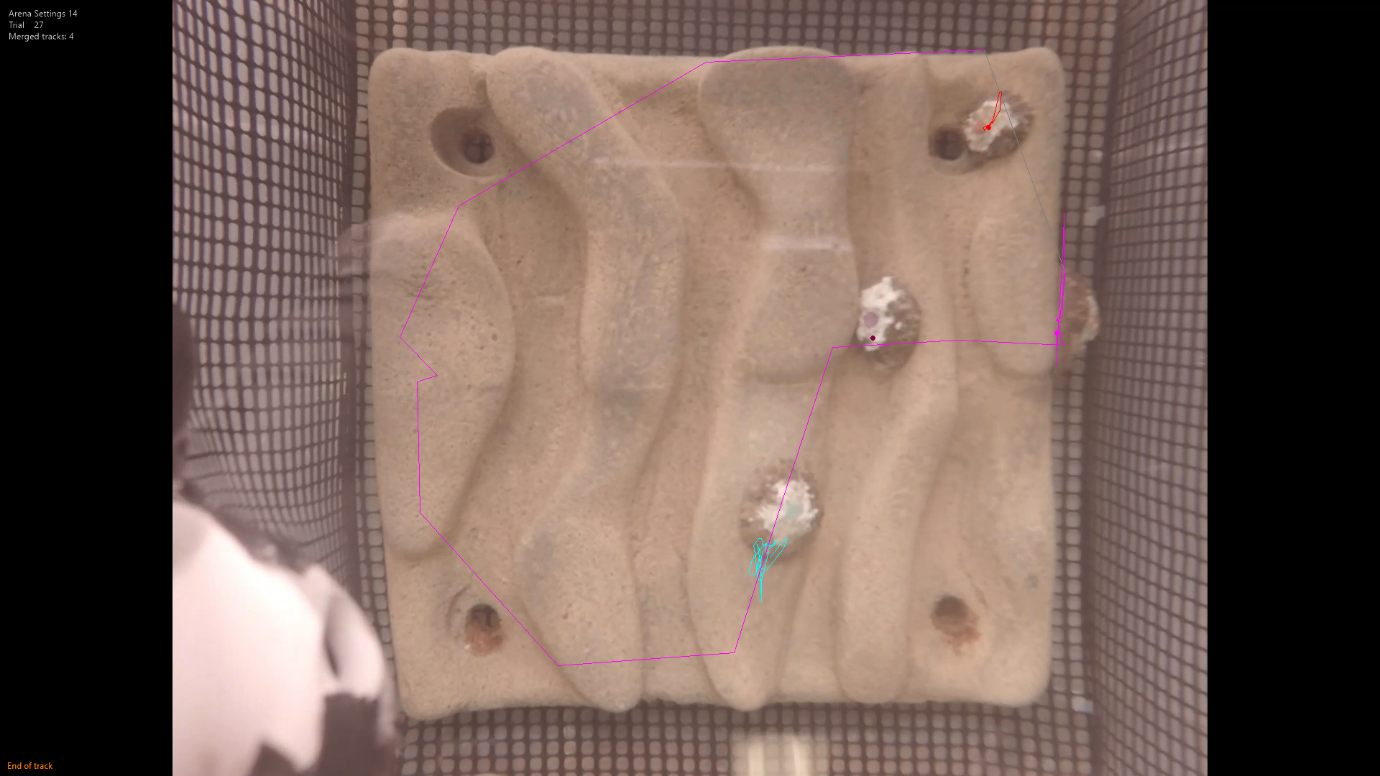
*

**Figure S5.** Example tracks of limpets derived from Ethovision XT (13.0) software for the 2.5 cm high panel. Each colour represents a single limpet. In the case of the dark purple limpet, no movement was recorded for this tidal/photoperiod combination (daytime high tide). These tracks were converted to xy coordinates for each frame of the video, from which distances were calculated.

**S1: Ethovision software calibration**

Arena definition (the region of the video image within which to track animals, here defined as the area of the panel) was set using still images from the time-lapse videos and calibrated using the height and width of the complexity panels. Detection settings (which determine how the animal will be detected within the video frames) were configured to detect the centre point of a limpet’s shell using dynamic subtraction, which compared an image with the previous image to detect changes in pixels representing animal movement (accounts for changes in background and lighting). Contour erosion (the maximum number of pixels making up the surface area of a limpet) was configured for each individual video to optimise detection of limpets. Video sample rate was set to 10 frames per second, corresponding to one sample for each photograph. Trial control settings (used to automate data collection) were set to start immediate data acquisition.

Following the acquisition of movement tracks, track smoothing (lowess) was set to smooth across five samples (two before and after every sample point) with the aim of eliminating ‘noise’ within the data. Additionally, a threshold distance for what was considered ‘true’ movement was set based on the average distance ‘moved’ per time step by five limpets that were identified as immobile for the duration of the 24-hour time lapse (threshold distance = 0.01 cm). Any movement recorded below this distance threshold was flagged as noise and the distance moved set to 0 cm. The resulting track profile for each limpet was manually checked for abnormalities (i.e. rogue points, misdetection), which were replaced via interpolation and visually verified.

**Table S1.** The surface areas (m^2^) of the control treatment, plus the 12 experimental model grids used to simulate limpet movement over a grid of 8 × 16 panels, representing a 2 × 4 m area.

| **Spatial cover** | **Panel** | **Surface area (m^2^)** |
| --- | --- | --- |
| Control (100%) | Flat | 7.68 |
| ~14% | Ripple | 8.04 |
| ~14% | 2.5 cm high | 8.22 |
| ~14% | 5 cm high | 8.40 |
| ~14% | 3-Panel | 8.22 |
| ~33% | Ripple | 8.52 |
| ~33% | 2.5 cm high | 8.94 |
| ~33% | 5 cm high | 9.36 |
| ~33% | 3-Panel | 8.94 |
| ~50% | Ripple | 8.94 |
| ~50% | 2.5 cm high | 9.57 |
| ~50% | 5 cm high | 10.2 |
| ~50% | 3-Panel | 9.57 |

**Table S2.** The results of post hoc tests for the effects of panel type on gross and net distance moved by limpets over a 24-hour period. *** indicates significance at the 0.005 level.

|  | **Contrast** | **Estimate** | **Std. Error** | **DoF** | **t-ratio** | **p-value** |
| --- | --- | --- | --- | --- | --- | --- |
| **Gross distance** | Flat ~ Ripple | -0.75 | 0.18 | 91 | -4.12 | < 0.001 |
|  | Flat ~ 2.5 cm high | 0.39 | 0.17 | 91 | 2.36 | 0.09 |
|  | Flat ~ 5 cm high | 0.07 | 0.16 | 91 | 0.46 | 0.97 |
|  | Ripple ~ 2.5 cm high | -0.36 | 0.18 | 91 | -2.01 | 0.19 |
|  | Ripple ~ 5 cm high | -0.68 | 0.18 | 91 | -3.71 | < 0.005 |
|  | 2.5 cm high ~ 5 cm high | 0.31 | 0.17 | 91 | 1.91 | 0.23 |
| **Net distance** | Flat ~ Ripple | -0.63 | 0.16 | 92 | -3.95 | < 0.005 |
|  | Flat ~ 2.5 cm high | 0.36 | 0.15 | 92 | 2.41 | 0.08 |
|  | Flat ~ 5 cm high | 0.23 | 0.15 | 92 | 1.55 | 0.42 |
|  | Ripple ~ 2.5 cm high | -0.27 | 0.16 | 92 | -1.73 | 0.32 |
|  | Ripple ~ 5 cm high | -0.40 | 0.16 | 92 | -2.50 | 0.07 |
|  | 2.5 cm high ~ 5 cm high | 0.13 | 0.15 | 92 | 0.85 | 0.83 |


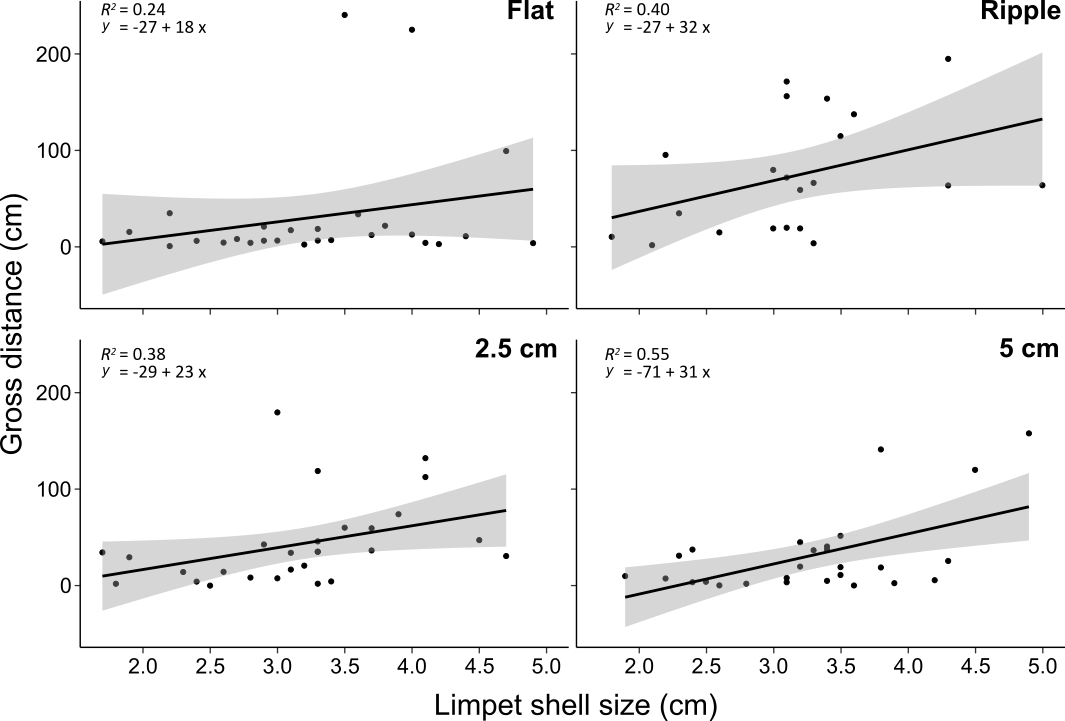


**Figure S6.** The effect of limpet shell length (cm) on gross distance (cm) moved by limpets on each panel type. Lines represent linear models, with shaded areas showing the 95% confidence intervals. Model equations and R^2^ values are shown alongside each plot.

**Table S3.** The results of post hoc tests for the effects of panel type and immersion state on the probability of limpet movement at each 5-minute time step over a 24-hour period. * indicates significance at the 0.05 level.

|  | **Contrast** | **Estimate** | **Std. error** | **DoF** | **t-ratio** | **p-value** |
| --- | --- | --- | --- | --- | --- | --- |
| **2.5 cm high: Immersed** | 5 cm high; Immersed | 0.20 | 0.25 | 91 | 0.80 | 0.99 |
|  | Flat; Immersed | -0.598 | 0.25 | 91 | -2.31 | 0.30 |
|  | Ripple; Immersed | -0.41 | 0.27 | 91 | -1.49 | 0.81 |
|  | 2.5 cm high; Emersed | 0.62 | 0.15 | 29037 | 4.16 | < 0.005 * |
|  | 5 cm high; Emersed | 0.47 | 0.25 | 91 | 1.88 | 0.57 |
|  | Flat; Emersed | 0.30 | 0.25 | 91 | 1.19 | 0.93 |
|  | Ripple; Emersed | 0.26 | 0.27 | 91 | 0.94 | 0.99 |
| **5 cm high; Emersed** | Flat; Immersed | -0.78 | 0.25 | 91 | -3.14 | 0.05 . |
|  | Ripple; Immersed | -0.60 | 0.28 | 91 | -2.20 | 0.36 |
|  | 2.5 cm high; Emersed | 0.42 | 0.25 | 91 | 1.69 | 0.70 |
|  | 5 cm high; Emersed | 0.27 | 0.15 | 29037 | 1.78 | 0.63 |
|  | Flat; Emersed | 0.10 | 0.25 | 91 | 0.40 | 1.0 |
|  | Ripple; Emersed | 0.06 | 0.28 | 91 | 0.21 | 1.0 |
| **Flat; Immersed** | Ripple; Immersed | 0.18 | 0.28 | 91 | 0.63 | 1.0 |
|  | 2.5 cm high; Emersed | 1.20 | 0.25 | 91 | 4.78 | < 0.001 * |
|  | 5 cm high; Emersed | 1.05 | 0.25 | 91 | 4.23 | < 0.005 * |
|  | Flat; Emersed | 0.88 | 0.16 | 29037 | 5.55 | < 0.001 * |
|  | Ripple; Emersed | 0.84 | 0.28 | 91 | 3.03 | 0.06 |
| **Ripple; Immersed** | 2.5 cm high; Emersed | 1.03 | 0.27 | 91 | 3.78 | 0.01 * |
|  | 5 cm high; Emersed | 0.88 | 0.28 | 91 | 3.18 | 0.04 * |
|  | Flat; Emersed | 0.70 | 0.28 | 91 | 2.55 | 0.19 |
|  | Ripple; Emersed | 0.66 | 0.17 | 29037 | 3.83 | < 0.005 * |
| **2.5 cm high; Emersed** | 5 cm high; Emersed | -0.15 | 0.25 | 91 | -0.60 | 1.0 |
|  | Flat; Emersed | -0.32 | 0.25 | 91 | -1.28 | 0.90 |
|  | Ripple; Emersed | -0.36 | 0.27 | 91 | -1.35 | 0.88 |
| **5 cm high; Emersed** | Flat; Emersed | -0.17 | 0.25 | 91 | -0.69 | 1.0 |
|  | Ripple; Emersed | -0.21 | 0.27 | 91 | -0.78 | 0.99 |
| **Flat; Emersed** | Ripple; Emersed | -0.04 | 0.28 | 91 | -0.15 | 1.0 |


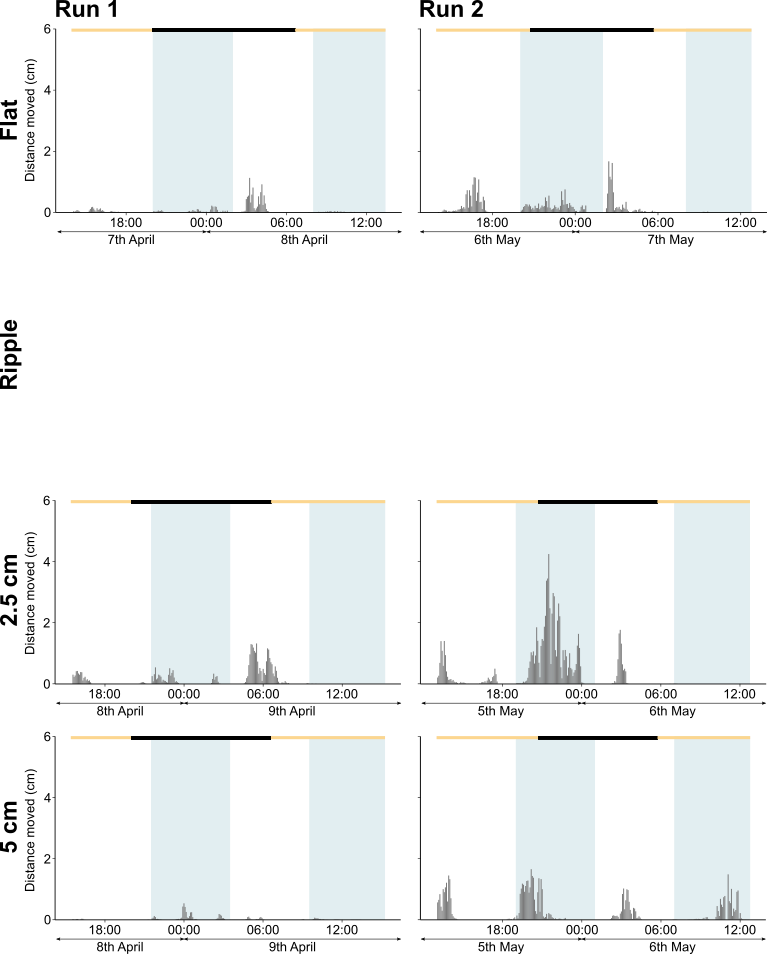


**Figure S7A.** For replicates 1 and 2, the average distance moved by limpets on flat, 2.5 cm high and 5 cm high panels for each time step (5-min) of the 24-hour time-lapse. At the top of each plot, periods of daylight are indicated by yellow bars and nocturnal periods by black bars. Blue shading indicates periods of immersion, which matched the actual tide times on the day of recording as closely as possible. Plots for the ripple panel are absent due to failed recording (e.g., due to camera or torch failures).


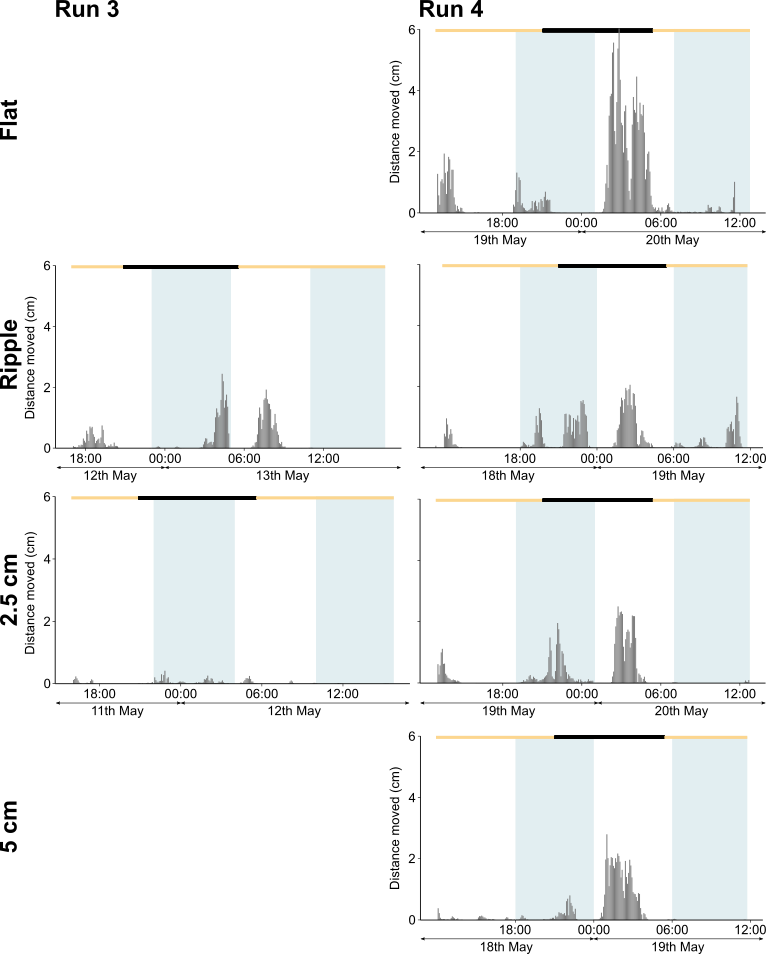


**Figure S7B.** For replicates 3 and 4, the average distance moved by limpets on flat, 2.5 cm high and 5 cm high panels for each time step (5-min) of the 24-hour time-lapse. At the top of each plot, periods of daylight are indicated by yellow bars and nocturnal periods by black bars. Blue shading indicates periods of immersion, which matched the actual tide times on the day of recording as closely as possible. Plots for the flat and 5 cm high panels are absent for replicate 3 due to failed recording (e.g., due to camera or torch failures).

**
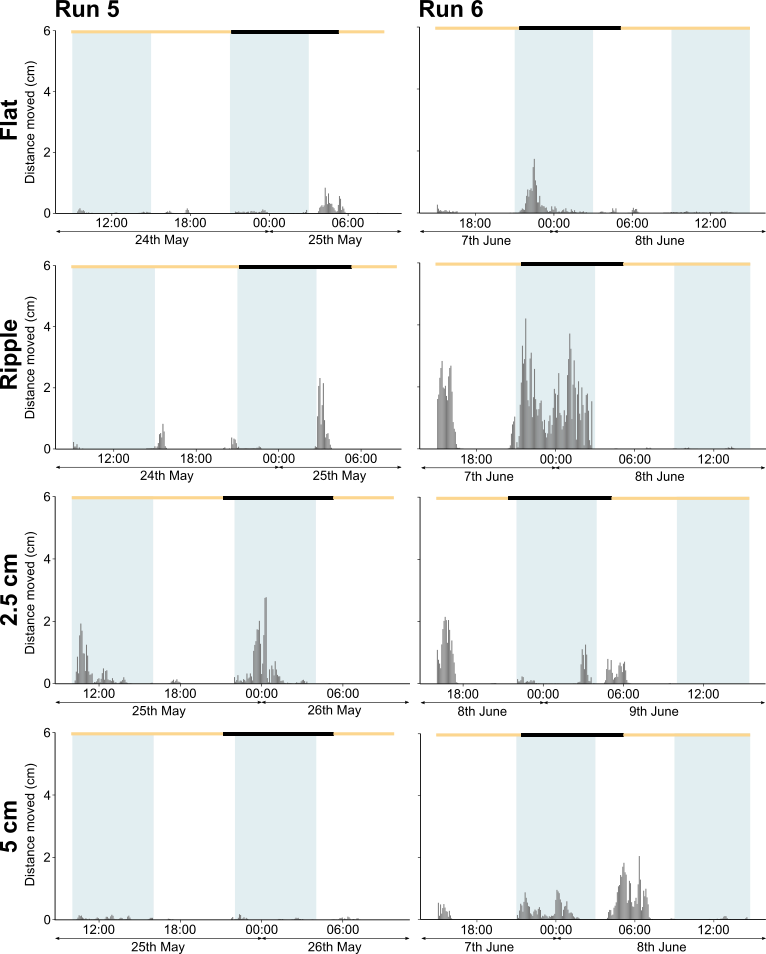
**

**FIGURE S7C.** For replicates 5 and 6, the average distance moved by limpets on flat, 2.5 cm high and 5 cm high panels for each time step (5-min) of the 24-hour time-lapse. At the top of each plot, periods of daylight are indicated by yellow bars and nocturnal periods by black bars. Blue shading indicates periods of immersion, which matched the actual tide times on the day of recording as closely as possible.


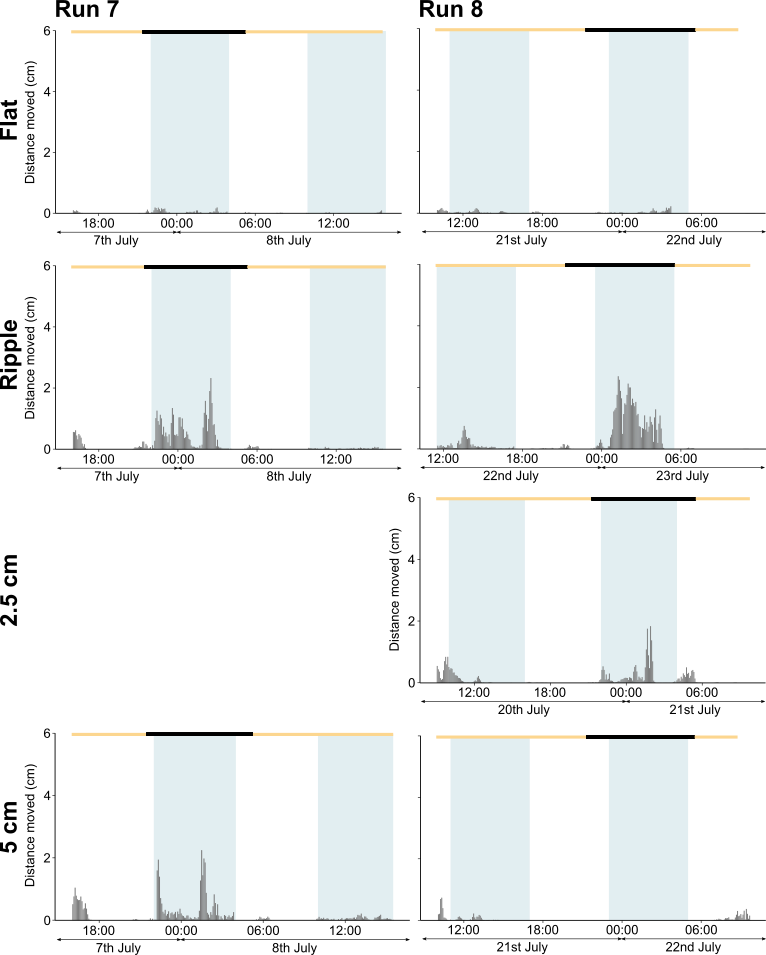


**FIGURE S7D.** For replicates 7 and 8, the average distance moved by limpets on flat, 2.5 cm high and 5 cm high panels for each time step (5-min) of the 24-hour time-lapse. At the top of each plot, periods of daylight are indicated by yellow bars and nocturnal periods by black bars. Blue shading indicates periods of immersion, which matched the actual tide times on the day of recording as closely as possible. Plots for the 2.5 cm high panel are absent for replicate 7 due to failed recording (e.g., due to camera or torch failures).

***
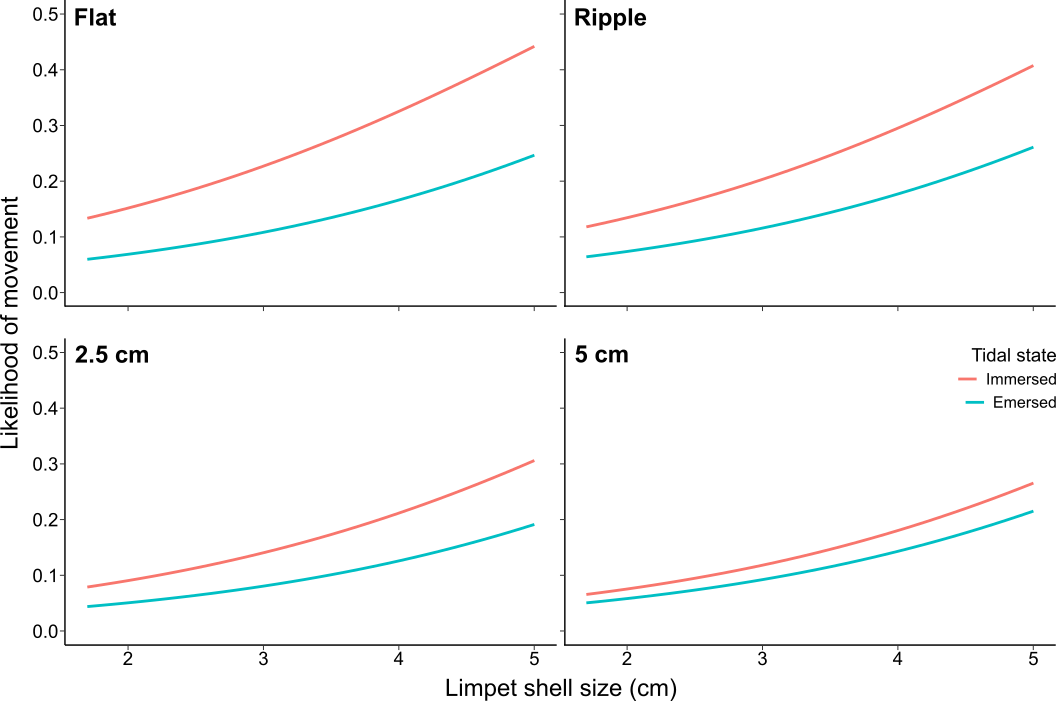
***

**Figure S8.** The predicted likelihood of limpet movement at increasing shell sizes (cm) for the four panel types during periods of immersion (pink) and emersion (blue).

**Table S4.** The results of post hoc tests for the effects of panel type (Ripple; 2.5 cm high; 5 cm high; 3 panel), spatial configuration (Grouped; Random) and area (14%; 33%; 50%) on the path lengths (cm) of 1000 simulated limpets.

|  | **Contrast** | **Estimate** | **SE** | **DoF** | **t-ratio** | **p-value** |
| --- | --- | --- | --- | --- | --- | --- |
| **Ripple – Grouped – 14%** | Control | 0.206 | 0.031 | 6993 | 6.714 | < 0.001 |
|  | 5 cm high – Grouped – 14% | 0.222 | 0.030 | 23976 | 7.389 | < 0.001 |
|  | 2.5 cm high – Grouped – 14% | -0.012 | 0.030 | 23976 | -0.390 | 1 |
|  | 3 panel – Grouped 14% | -0.005 | 0.030 | 23976 | -0.182 | 1 |
|  | Ripple – Random – 14% | -0.184 | 0.030 | 23976 | -6.122 | < 0.001 |
|  | 5 cm high – Random – 14% | 0.389 | 0.030 | 23976 | 12.965 | < 0.001 |
|  | 2.5 cm high – Random – 14% | -0.007 | 0.030 | 23976 | -0.231 | 1 |
|  | 3 panel – Random – 14% | 0.019 | 0.030 | 23976 | 0.621 | 1 |
|  | Ripple – Grouped – 33% | -0.377 | 0.030 | 23976 | -12.540 | < 0.001 |
|  | 5 cm high – Grouped – 33% | 0.517 | 0.030 | 23976 | 17.209 | < 0.001 |
|  | 2.5 cm high – Grouped – 33% | -0.242 | 0.030 | 23976 | -8.042 | < 0.001 |
|  | 3 panel – Grouped 33% | -0.087 | 0.030 | 23976 | -2.899 | 0.363 |
|  | Ripple – Random – 33% | -0.566 | 0.030 | 23976 | -18.846 | < 0.001 |
|  | 5 cm high – Random – 33% | 0.288 | 0.030 | 23976 | 9.576 | < 0.001 |
|  | 2.5 cm high – Random – 33% | -0.416 | 0.030 | 23976 | -13.839 | < 0.001 |
|  | 3 panel – Random – 33% | -0.274 | 0.030 | 23976 | -9.135 | < 0.001 |
|  | Ripple – Grouped – 50% | -0.562 | 0.030 | 23976 | -18.716 | < 0.001 |
|  | 5 cm high – Grouped – 50% | 0.397 | 0.030 | 23976 | 13.224 | < 0.001 |
|  | 2.5 cm high – Grouped – 50% | -0.468 | 0.030 | 23976 | -15.587 | < 0.001 |
|  | 3 panel – Grouped 50% | -0.276 | 0.030 | 23976 | -9.192 | < 0.001 |
|  | Ripple – Random – 50% | -0.778 | 0.030 | 23976 | -25.886 | < 0.001 |
|  | 5 cm high – Random – 50% | 0.284 | 0.030 | 23976 | 9.449 | < 0.001 |
|  | 2.5 cm high – Random – 50% | -0.500 | 0.030 | 23976 | -16.650 | < 0.001 |
|  | 3 panel – Random – 50% | -0.453 | 0.030 | 23976 | -15.096 | < 0.001 |
| **5 cm high – Grouped – 14%** | Control | -0.016 | 0.028 | 6993 | -0.572 | 0.567 |
|  | 2.5 cm high – Grouped – 14% | -0.234 | 0.030 | 23976 | -7.779 | < 0.001 |
|  | 3 panel – Grouped 14% | -0.227 | 0.030 | 23976 | -7.571 | < 0.001 |
|  | Ripple – Random – 14% | -0.406 | 0.030 | 23976 | -13.511 | < 0.001 |
|  | 5 cm high – Random – 14% | 0.168 | 0.030 | 23976 | 5.575 | < 0.001 |
|  | 2.5 cm high – Random – 14% | -0.229 | 0.030 | 23976 | -7.620 | < 0.001 |
|  | 3 panel – Random – 14% | -0.203 | 0.030 | 23976 | -6.768 | < 0.001 |
|  | Ripple – Grouped – 33% | -0.599 | 0.030 | 23976 | -19.929 | < 0.001 |
|  | 5 cm high – Grouped – 33% | 0.295 | 0.030 | 23976 | 9.820 | < 0.001 |
|  | 2.5 cm high – Grouped – 33% | -0.464 | 0.030 | 23976 | -15.431 | < 0.001 |
|  | 3 panel – Grouped 33% | -0.309 | 0.030 | 23976 | -10.288 | < 0.001 |
|  | Ripple – Random – 33% | -0.788 | 0.030 | 23976 | -26.236 | < 0.001 |
|  | 5 cm high – Random – 33% | 0.066 | 0.030 | 23976 | 2.187 | 0.877 |
|  | 2.5 cm high – Random – 33% | -0.638 | 0.030 | 23976 | -21.228 | < 0.001 |
|  | 3 panel – Random – 33% | -0.496 | 0.030 | 23976 | -16.524 | < 0.001 |
|  | Ripple – Grouped – 50% | -0.784 | 0.030 | 23976 | -26.105 | < 0.001 |
|  | 5 cm high – Grouped – 50% | 0.175 | 0.030 | 23976 | 5.835 | < 0.001 |
|  | 2.5 cm high – Grouped – 50% | -0.690 | 0.030 | 23976 | -22.976 | < 0.001 |
|  | 3 panel – Grouped 50% | -0.498 | 0.030 | 23976 | -16.582 | < 0.001 |
|  | Ripple – Random – 50% | -1.000 | 0.030 | 23976 | -33.275 | < 0.001 |
|  | 5 cm high – Random – 50% | 0.062 | 0.030 | 23976 | 2.060 | 0.929 |
|  | 2.5 cm high – Random – 50% | -0.722 | 0.030 | 23976 | -24.039 | < 0.001 |
|  | 3 panel – Random – 50% | -0.676 | 0.030 | 23976 | -22.485 | < 0.001 |
| **2.5 cm high – Grouped – 14%** | Control | 0.217 | 0.029 | 6993 | 7.380 | < 0.001 |
|  | 3 panel – Grouped 14% | 0.006 | 0.030 | 23976 | 0.208 | 1 |
|  | Ripple – Random – 14% | -0.172 | 0.030 | 23976 | -5.732 | < 0.001 |
|  | 5 cm high – Random – 14% | 0.401 | 0.030 | 23976 | 13.355 | < 0.001 |
|  | 2.5 cm high – Random – 14% | 0.005 | 0.030 | 23976 | 0.159 | 1 |
|  | 3 panel – Random – 14% | 0.030 | 0.030 | 23976 | 1.011 | 1 |
|  | Ripple – Grouped – 33% | -0.365 | 0.030 | 23976 | -12.150 | < 0.001 |
|  | 5 cm high – Grouped – 33% | 0.529 | 0.030 | 23976 | 17.600 | < 0.001 |
|  | 2.5 cm high – Grouped – 33% | -0.230 | 0.030 | 23976 | -7.651 | < 0.001 |
|  | 3 panel – Grouped 33% | -0.075 | 0.030 | 23976 | -2.508 | 0.670 |
|  | Ripple – Random – 33% | -0.554 | 0.030 | 23976 | -18.456 | < 0.001 |
|  | 5 cm high – Random – 33% | 0.299 | 0.030 | 23976 | 9.966 | < 0.001 |
|  | 2.5 cm high – Random – 33% | -0.404 | 0.030 | 23976 | -13.449 | < 0.001 |
|  | 3 panel – Random – 33% | -0.263 | 0.030 | 23976 | -8.744 | < 0.001 |
|  | Ripple – Grouped – 50% | -0.551 | 0.030 | 23976 | -18.326 | < 0.001 |
|  | 5 cm high – Grouped – 50% | 0.409 | 0.030 | 23976 | 13.614 | < 0.001 |
|  | 2.5 cm high – Grouped – 50% | -0.457 | 0.030 | 23976 | -15.197 | < 0.001 |
|  | 3 panel – Grouped 50% | -0.264 | 0.030 | 23976 | -8.802 | < 0.001 |
|  | Ripple – Random – 50% | -0.766 | 0.030 | 23976 | -25.496 | < 0.001 |
|  | 5 cm high – Random – 50% | 0.296 | 0.030 | 23976 | 9.839 | < 0.001 |
|  | 2.5 cm high – Random – 50% | -0.488 | 0.030 | 23976 | -16.260 | < 0.001 |
|  | 3 panel – Random – 50% | -0.442 | 0.030 | 23976 | -14.706 | < 0.001 |
| **3 panel – Grouped – 14%** | Control | 0.211 | 0.030 | 6993 | 6.997 | < 0.001 |
|  | Ripple – Random – 14% | -0.178 | 0.030 | 23976 | -5.939 | < 0.001 |
|  | 5 cm high – Random – 14% | 0.395 | 0.030 | 23976 | 13.147 | < 0.001 |
|  | 2.5 cm high – Random – 14% | -0.001 | 0.030 | 23976 | -0.049 | 1 |
|  | 3 panel – Random – 14% | 0.024 | 0.030 | 23976 | 0.803 | 1 |
|  | Ripple – Grouped – 33% | -0.371 | 0.030 | 23976 | -12.358 | < 0.001 |
|  | 5 cm high – Grouped – 33% | 0.522 | 0.030 | 23976 | 17.392 | < 0.001 |
|  | 2.5 cm high – Grouped – 33% | -0.236 | 0.030 | 23976 | -7.859 | < 0.001 |
|  | 3 panel – Grouped 33% | -0.082 | 0.030 | 23976 | -2.716 | 0.503 |
|  | Ripple – Random – 33% | -0.561 | 0.030 | 23976 | -18.664 | < 0.001 |
|  | 5 cm high – Random – 33% | 0.293 | 0.030 | 23976 | 9.758 | < 0.001 |
|  | 2.5 cm high – Random – 33% | -0.410 | 0.030 | 23976 | -13.657 | < 0.001 |
|  | 3 panel – Random – 33% | -0.269 | 0.030 | 23976 | -8.952 | < 0.001 |
|  | Ripple – Grouped – 50% | -0.557 | 0.030 | 23976 | -18.534 | < 0.001 |
|  | 5 cm high – Grouped – 50% | 0.403 | 0.030 | 23976 | 13.406 | < 0.001 |
|  | 2.5 cm high – Grouped – 50% | -0.463 | 0.030 | 23976 | -15.405 | < 0.001 |
|  | 3 panel – Grouped 50% | -0.271 | 0.030 | 23976 | -9.010 | < 0.001 |
|  | Ripple – Random – 50% | -0.772 | 0.030 | 23976 | -25.704 | < 0.001 |
|  | 5 cm high – Random – 50% | 0.289 | 0.030 | 23976 | 9.631 | < 0.001 |
|  | 2.5 cm high – Random – 50% | -0.495 | 0.030 | 23976 | -16.467 | < 0.001 |
|  | 3 panel – Random – 50% | -0.448 | 0.030 | 23976 | -14.914 | < 0.001 |
| **Ripple – Random – 14%** | Control | 0.390 | 0.031 | 6993 | 12.717 | < 0.001 |
|  | 5 cm high – Random – 14% | 0.573 | 0.030 | 23976 | 19.086 | < 0.001 |
|  | 2.5 cm high – Random – 14% | 0.177 | 0.030 | 23976 | 5.891 | < 0.001 |
|  | 3 panel – Random – 14% | 0.203 | 0.030 | 23976 | 6.743 | < 0.001 |
|  | Ripple – Grouped – 33% | -0.193 | 0.030 | 23976 | -6.418 | < 0.001 |
|  | 5 cm high – Grouped – 33% | 0.701 | 0.030 | 23976 | 23.331 | < 0.001 |
|  | 2.5 cm high – Grouped – 33% | -0.058 | 0.030 | 23976 | -1.920 | 0.966 |
|  | 3 panel – Grouped 33% | 0.097 | 0.030 | 23976 | 3.223 | 0.172 |
|  | Ripple – Random – 33% | -0.382 | 0.030 | 23976 | -12.725 | < 0.001 |
|  | 5 cm high – Random – 33% | 0.472 | 0.030 | 23976 | 15.697 | < 0.001 |
|  | 2.5 cm high – Random – 33% | -0.232 | 0.030 | 23976 | -7.717 | < 0.001 |
|  | 3 panel – Random – 33% | -0.091 | 0.030 | 23976 | -3.013 | 0.286 |
|  | Ripple – Grouped – 50% | -0.378 | 0.030 | 23976 | -12.594 | < 0.001 |
|  | 5 cm high – Grouped – 50% | 0.581 | 0.030 | 23976 | 19.346 | < 0.001 |
|  | 2.5 cm high – Grouped – 50% | -0.284 | 0.030 | 23976 | -9.465 | < 0.001 |
|  | 3 panel – Grouped 50% | -0.092 | 0.030 | 23976 | -3.071 | 0.251 |
|  | Ripple – Random – 50% | -0.594 | 0.030 | 23976 | -19.764 | < 0.001 |
|  | 5 cm high – Random – 50% | 0.468 | 0.030 | 23976 | 15.571 | < 0.001 |
|  | 2.5 cm high – Random – 50% | -0.316 | 0.030 | 23976 | -10.528 | < 0.001 |
|  | 3 panel – Random – 50% | -0.270 | 0.030 | 23976 | -8.974 | < 0.001 |
| **5 cm high – Random – 14%** | Control | -0.184 | 0.028 | 6993 | -6.454 | < 0.001 |
|  | 2.5 cm high – Random – 14% | -0.396 | 0.030 | 23976 | -13.196 | < 0.001 |
|  | 3 panel – Random – 14% | -0.371 | 0.030 | 23976 | -12.344 | < 0.001 |
|  | Ripple – Grouped – 33% | -0.766 | 0.030 | 23976 | -25.505 | < 0.001 |
|  | 5 cm high – Grouped – 33% | 0.128 | 0.030 | 23976 | 4.245 | 0.005 |
|  | 2.5 cm high – Grouped – 33% | -0.631 | 0.030 | 23976 | -21.006 | < 0.001 |
|  | 3 panel – Grouped 33% | -0.477 | 0.030 | 23976 | -15.863 | < 0.001 |
|  | Ripple – Random – 33% | -0.956 | 0.030 | 23976 | -31.811 | < 0.001 |
|  | 5 cm high – Random – 33% | -0.102 | 0.030 | 23976 | -3.389 | 0.109 |
|  | 2.5 cm high – Random – 33% | -0.805 | 0.030 | 23976 | -26.804 | < 0.001 |
|  | 3 panel – Random – 33% | -0.664 | 0.030 | 23976 | -22.099 | < 0.001 |
|  | Ripple – Grouped – 50% | -0.952 | 0.030 | 23976 | -31.681 | < 0.001 |
|  | 5 cm high – Grouped – 50% | 0.008 | 0.030 | 23976 | 0.259 | 1 |
|  | 2.5 cm high – Grouped – 50% | -0.858 | 0.030 | 23976 | -28.551 | < 0.001 |
|  | 3 panel – Grouped 50% | -0.666 | 0.030 | 23976 | -22.157 | < 0.001 |
|  | Ripple – Random – 50% | -1.167 | 0.030 | 23976 | -38.851 | < 0.001 |
|  | 5 cm high – Random – 50% | -0.106 | 0.030 | 23976 | -3.516 | 0.074 |
|  | 2.5 cm high – Random – 50% | -0.890 | 0.030 | 23976 | -29.614 | < 0.001 |
|  | 3 panel – Random – 50% | -0.843 | 0.030 | 23976 | -28.061 | < 0.001 |
| **2.5 cm high – Random – 14%** | Control | 0.213 | 0.029 | 6993 | 7.218 | < 0.001 |
|  | 3 panel – Random – 14% | 0.026 | 0.030 | 23976 | 0.852 | 1 |
|  | Ripple – Grouped – 33% | -0.370 | 0.030 | 23976 | -12.309 | < 0.001 |
|  | 5 cm high – Grouped – 33% | 0.524 | 0.030 | 23976 | 17.440 | < 0.001 |
|  | 2.5 cm high – Grouped – 33% | -0.235 | 0.030 | 23976 | -7.811 | < 0.001 |
|  | 3 panel – Grouped 33% | -0.080 | 0.030 | 23976 | -2.668 | 0.542 |
|  | Ripple – Random – 33% | -0.559 | 0.030 | 23976 | -18.615 | < 0.001 |
|  | 5 cm high – Random – 33% | 0.295 | 0.030 | 23976 | 9.807 | < 0.001 |
|  | 2.5 cm high – Random – 33% | -0.409 | 0.030 | 23976 | -13.608 | < 0.001 |
|  | 3 panel – Random – 33% | -0.268 | 0.030 | 23976 | -8.904 | < 0.001 |
|  | Ripple – Grouped – 50% | -0.555 | 0.030 | 23976 | -18.485 | < 0.001 |
|  | 5 cm high – Grouped – 50% | 0.404 | 0.030 | 23976 | 13.455 | < 0.001 |
|  | 2.5 cm high – Grouped – 50% | -0.461 | 0.030 | 23976 | -15.356 | < 0.001 |
|  | 3 panel – Grouped 50% | -0.269 | 0.030 | 23976 | -8.961 | < 0.001 |
|  | Ripple – Random – 50% | -0.771 | 0.030 | 23976 | -25.655 | < 0.001 |
|  | 5 cm high – Random – 50% | 0.291 | 0.030 | 23976 | 9.680 | < 0.001 |
|  | 2.5 cm high – Random – 50% | -0.493 | 0.030 | 23976 | -16.419 | < 0.001 |
|  | 3 panel – Random – 50% | -0.447 | 0.030 | 23976 | -14.865 | < 0.001 |
| **3 panel – Random – 14%** | Control | 0.187 | 0.030 | 6993 | 6.197 | < 0.001 |
|  | Ripple – Grouped – 33% | -0.395 | 0.030 | 23976 | -13.161 | < 0.001 |
|  | 5 cm high – Grouped – 33% | 0.498 | 0.030 | 23976 | 16.588 | < 0.001 |
|  | 2.5 cm high – Grouped – 33% | -0.260 | 0.030 | 23976 | -8.663 | < 0.001 |
|  | 3 panel – Grouped 33% | -0.106 | 0.030 | 23976 | -3.520 | 0.073 |
|  | Ripple – Random – 33% | -0.585 | 0.030 | 23976 | -19.468 | < 0.001 |
|  | 5 cm high – Random – 33% | 0.269 | 0.030 | 23976 | 8.955 | < 0.001 |
|  | 2.5 cm high – Random – 33% | -0.434 | 0.030 | 23976 | -14.460 | < 0.001 |
|  | 3 panel – Random – 33% | -0.293 | 0.030 | 23976 | -9.756 | < 0.001 |
|  | Ripple – Grouped – 50% | -0.581 | 0.030 | 23976 | -19.337 | < 0.001 |
|  | 5 cm high – Grouped – 50% | 0.379 | 0.030 | 23976 | 12.603 | < 0.001 |
|  | 2.5 cm high – Grouped – 50% | -0.487 | 0.030 | 23976 | -16.208 | < 0.001 |
|  | 3 panel – Grouped 50% | -0.295 | 0.030 | 23976 | -9.813 | < 0.001 |
|  | Ripple – Random – 50% | -0.796 | 0.030 | 23976 | -26.507 | < 0.001 |
|  | 5 cm high – Random – 50% | 0.265 | 0.030 | 23976 | 8.828 | < 0.001 |
|  | 2.5 cm high – Random – 50% | -0.519 | 0.030 | 23976 | -17.271 | < 0.001 |
|  | 3 panel – Random – 50% | -0.472 | 0.030 | 23976 | -15.717 | < 0.001 |
| **Ripple – Grouped – 33%** | Control | 0.582 | 0.031 | 6993 | 19.011 | < 0.001 |
|  | 5 cm high – Grouped – 33% | 0.894 | 0.030 | 23976 | 29.750 | < 0.001 |
|  | 2.5 cm high – Grouped – 33% | 0.135 | 0.030 | 23976 | 4.499 | 0.002 |
|  | 3 panel – Grouped 33% | 0.290 | 0.030 | 23976 | 9.642 | < 0.001 |
|  | Ripple – Random – 33% | -0.189 | 0.030 | 23976 | -6.306 | < 0.001 |
|  | 5 cm high – Random – 33% | 0.664 | 0.030 | 23976 | 22.116 | < 0.001 |
|  | 2.5 cm high – Random – 33% | -0.039 | 0.030 | 23976 | -1.299 | 1 |
|  | 3 panel – Random – 33% | 0.102 | 0.030 | 23976 | 3.405 | 0.104 |
|  | Ripple – Grouped – 50% | -0.186 | 0.030 | 23976 | -6.176 | < 0.001 |
|  | 5 cm high – Grouped – 50% | 0.774 | 0.030 | 23976 | 25.764 | < 0.001 |
|  | 2.5 cm high – Grouped – 50% | -0.092 | 0.030 | 23976 | -3.047 | 0.265 |
|  | 3 panel – Grouped 50% | 0.101 | 0.030 | 23976 | 3.348 | 0.122 |
|  | Ripple – Random – 50% | -0.401 | 0.030 | 23976 | -13.346 | < 0.001 |
|  | 5 cm high – Random – 50% | 0.661 | 0.030 | 23976 | 21.989 | < 0.001 |
|  | 2.5 cm high – Random – 50% | -0.123 | 0.030 | 23976 | -4.109 | 0.009 |
|  | 3 panel – Random – 50% | -0.077 | 0.030 | 23976 | -2.556 | 0.633 |
| **5 cm high – Grouped – 33%** | Control | -0.311 | 0.028 | 6993 | -10.931 | < 0.001 |
|  | 2.5 cm high – Grouped – 33% | -0.759 | 0.030 | 23976 | -25.251 | < 0.001 |
|  | 3 panel – Grouped 33% | -0.604 | 0.030 | 23976 | -20.108 | < 0.001 |
|  | Ripple – Random – 33% | -1.083 | 0.030 | 23976 | -36.056 | < 0.001 |
|  | 5 cm high – Random – 33% | -0.229 | 0.030 | 23976 | -7.634 | < 0.001 |
|  | 2.5 cm high – Random – 33% | -0.933 | 0.030 | 23976 | -31.049 | < 0.001 |
|  | 3 panel – Random – 33% | -0.791 | 0.030 | 23976 | -26.344 | < 0.001 |
|  | Ripple – Grouped – 50% | -1.079 | 0.030 | 23976 | -35.925 | < 0.001 |
|  | 5 cm high – Grouped – 50% | -0.120 | 0.030 | 23976 | -3.986 | 0.014 |
|  | 2.5 cm high – Grouped – 50% | -0.985 | 0.030 | 23976 | -32.796 | < 0.001 |
|  | 3 panel – Grouped 50% | -0.793 | 0.030 | 23976 | -26.402 | < 0.001 |
|  | Ripple – Random – 50% | -1.295 | 0.030 | 23976 | -43.095 | < 0.001 |
|  | 5 cm high – Random – 50% | -0.233 | 0.030 | 23976 | -7.760 | < 0.001 |
|  | 2.5 cm high – Random – 50% | -1.017 | 0.030 | 23976 | -33.859 | < 0.001 |
|  | 3 panel – Random – 50% | -0.971 | 0.030 | 23976 | -32.305 | < 0.001 |
| **2.5 cm high – Grouped – 33%** | Control | 0.447 | 0.029 | 6993 | 15.183 | < 0.001 |
|  | 3 panel – Grouped 33% | 0.155 | 0.030 | 23976 | 5.143 | < 0.001 |
|  | Ripple – Random – 33% | -0.325 | 0.030 | 23976 | -10.805 | < 0.001 |
|  | 5 cm high – Random – 33% | 0.529 | 0.030 | 23976 | 17.617 | < 0.001 |
|  | 2.5 cm high – Random – 33% | -0.174 | 0.030 | 23976 | -5.798 | < 0.001 |
|  | 3 panel – Random – 33% | -0.033 | 0.030 | 23976 | -1.093 | 1 |
|  | Ripple – Grouped – 50% | -0.321 | 0.030 | 23976 | -10.674 | < 0.001 |
|  | 5 cm high – Grouped – 50% | 0.639 | 0.030 | 23976 | 21.265 | < 0.001 |
|  | 2.5 cm high – Grouped – 50% | -0.227 | 0.030 | 23976 | -7.545 | < 0.001 |
|  | 3 panel – Grouped 50% | -0.035 | 0.030 | 23976 | -1.151 | 1 |
|  | Ripple – Random – 50% | -0.536 | 0.030 | 23976 | -17.844 | < 0.001 |
|  | 5 cm high – Random – 50% | 0.525 | 0.030 | 23976 | 17.491 | < 0.001 |
|  | 2.5 cm high – Random – 50% | -0.259 | 0.030 | 23976 | -8.608 | < 0.001 |
|  | 3 panel – Random – 50% | -0.212 | 0.030 | 23976 | -7.054 | < 0.001 |
| **3 panel – Grouped – 33%** | Control | 0.293 | 0.030 | 6993 | 9.701 | < 0.001 |
|  | Ripple – Random – 33% | -0.479 | 0.030 | 23976 | -15.948 | < 0.001 |
|  | 5 cm high – Random – 33% | 0.375 | 0.030 | 23976 | 12.474 | < 0.001 |
|  | 2.5 cm high – Random – 33% | -0.329 | 0.030 | 23976 | -10.941 | < 0.001 |
|  | 3 panel – Random – 33% | -0.187 | 0.030 | 23976 | -6.236 | < 0.001 |
|  | Ripple – Grouped – 50% | -0.475 | 0.030 | 23976 | -15.817 | < 0.001 |
|  | 5 cm high – Grouped – 50% | 0.484 | 0.030 | 23976 | 16.122 | < 0.001 |
|  | 2.5 cm high – Grouped – 50% | -0.381 | 0.030 | 23976 | -12.688 | < 0.001 |
|  | 3 panel – Grouped 50% | -0.189 | 0.030 | 23976 | -6.294 | < 0.001 |
|  | Ripple – Random – 50% | -0.691 | 0.030 | 23976 | -22.987 | < 0.001 |
|  | 5 cm high – Random – 50% | 0.371 | 0.030 | 23976 | 12.348 | < 0.001 |
|  | 2.5 cm high – Random – 50% | -0.413 | 0.030 | 23976 | -13.751 | < 0.001 |
|  | 3 panel – Random – 50% | -0.366 | 0.030 | 23976 | -12.197 | < 0.001 |
| **Ripple – Random – 33%** | Control | 0.772 | 0.031 | 6993 | 25.195 | < 0.001 |
|  | 5 cm high – Random – 33% | 0.854 | 0.030 | 23976 | 28.422 | < 0.001 |
|  | 2.5 cm high – Random – 33% | 0.150 | 0.030 | 23976 | 5.007 | < 0.001 |
|  | 3 panel – Random – 33% | 0.292 | 0.030 | 23976 | 9.712 | < 0.001 |
|  | Ripple – Grouped – 50% | 0.004 | 0.030 | 23976 | 0.130 | 1 |
|  | 5 cm high – Grouped – 50% | 0.963 | 0.030 | 23976 | 32.070 | < 0.001 |
|  | 2.5 cm high – Grouped – 50% | 0.098 | 0.030 | 23976 | 3.260 | 0.156 |
|  | 3 panel – Grouped 50% | 0.290 | 0.030 | 23976 | 9.654 | < 0.001 |
|  | Ripple – Random – 50% | -0.211 | 0.030 | 23976 | -7.039 | < 0.001 |
|  | 5 cm high – Random – 50% | 0.850 | 0.030 | 23976 | 28.295 | < 0.001 |
|  | 2.5 cm high – Random – 50% | 0.066 | 0.030 | 23976 | 2.197 | 0.872 |
|  | 3 panel – Random – 50% | 0.113 | 0.030 | 23976 | 3.751 | 0.034 |
| **5 cm high – Random – 33%** | Control | -0.082 | 0.028 | 6993 | -2.879 | 0.004 |
|  | 2.5 cm high – Random – 33% | -0.703 | 0.030 | 23976 | -23.415 | < 0.001 |
|  | 3 panel – Random – 33% | -0.562 | 0.030 | 23976 | -18.710 | < 0.001 |
|  | Ripple – Grouped – 50% | -0.850 | 0.030 | 23976 | -28.292 | < 0.001 |
|  | 5 cm high – Grouped – 50% | 0.110 | 0.030 | 23976 | 3.648 | 0.048 |
|  | 2.5 cm high – Grouped – 50% | -0.756 | 0.030 | 23976 | -25.163 | < 0.001 |
|  | 3 panel – Grouped 50% | -0.564 | 0.030 | 23976 | -18.768 | < 0.001 |
|  | Ripple – Random – 50% | -1.065 | 0.030 | 23976 | -35.462 | < 0.001 |
|  | 5 cm high – Random – 50% | -0.004 | 0.030 | 23976 | -0.127 | 1 |
|  | 2.5 cm high – Random – 50% | -0.788 | 0.030 | 23976 | -26.225 | < 0.001 |
|  | 3 panel – Random – 50% | -0.741 | 0.030 | 23976 | -24.672 | < 0.001 |
| **2.5 cm high – Random – 33%** | Control | 0.621 | 0.029 | 6993 | 21.095 | < 0.001 |
|  | 3 panel – Random – 33% | 0.141 | 0.030 | 23976 | 4.704 | 0.001 |
|  | Ripple – Grouped – 50% | -0.147 | 0.030 | 23976 | -4.877 | < 0.001 |
|  | 5 cm high – Grouped – 50% | 0.813 | 0.030 | 23976 | 27.063 | < 0.001 |
|  | 2.5 cm high – Grouped – 50% | -0.053 | 0.030 | 23976 | -1.748 | 0.989 |
|  | 3 panel – Grouped 50% | 0.140 | 0.030 | 23976 | 4.647 | 0.001 |
|  | Ripple – Random – 50% | -0.362 | 0.030 | 23976 | -12.047 | < 0.001 |
|  | 5 cm high – Random – 50% | 0.700 | 0.030 | 23976 | 23.288 | < 0.001 |
|  | 2.5 cm high – Random – 50% | -0.084 | 0.030 | 23976 | -2.811 | 0.429 |
|  | 3 panel – Random – 50% | -0.038 | 0.030 | 23976 | -1.257 | 1 |
| **3 panel – Random – 33%** | Control | 0.480 | 0.030 | 6993 | 15.909 | < 0.001 |
|  | Ripple – Grouped – 50% | -0.288 | 0.030 | 23976 | -9.581 | < 0.001 |
|  | 5 cm high – Grouped – 50% | 0.672 | 0.030 | 23976 | 22.359 | < 0.001 |
|  | 2.5 cm high – Grouped – 50% | -0.194 | 0.030 | 23976 | -6.452 | < 0.001 |
|  | 3 panel – Grouped 50% | -0.002 | 0.030 | 23976 | -0.058 | 1 |
|  | Ripple – Random – 50% | -0.503 | 0.030 | 23976 | -16.751 | < 0.001 |
|  | 5 cm high – Random – 50% | 0.558 | 0.030 | 23976 | 18.584 | < 0.001 |
|  | 2.5 cm high – Random – 50% | -0.226 | 0.030 | 23976 | -7.515 | < 0.001 |
|  | 3 panel – Random – 50% | -0.179 | 0.030 | 23976 | -5.961 | < 0.001 |
| **Ripple – Grouped – 50%** | Control | 0.768 | 0.031 | 6993 | 25.067 | < 0.001 |
|  | 5 cm high – Grouped – 50% | 0.960 | 0.030 | 23976 | 31.940 | < 0.001 |
|  | 2.5 cm high – Grouped – 50% | 0.094 | 0.030 | 23976 | 3.129 | 0.218 |
|  | 3 panel – Grouped 50% | 0.286 | 0.030 | 23976 | 9.524 | < 0.001 |
|  | Ripple – Random – 50% | -0.215 | 0.030 | 23976 | -7.170 | < 0.001 |
|  | 5 cm high – Random – 50% | 0.846 | 0.030 | 23976 | 28.165 | < 0.001 |
|  | 2.5 cm high – Random – 50% | 0.062 | 0.030 | 23976 | 2.066 | 0.927 |
|  | 3 panel – Random – 50% | 0.109 | 0.030 | 23976 | 3.620 | 0.053 |
| **5 cm high – Grouped – 50%** | Control | -0.192 | 0.028 | 6993 | -6.727 | < 0.001 |
|  | 2.5 cm high – Grouped – 50% | -0.866 | 0.030 | 23976 | -28.811 | < 0.001 |
|  | 3 panel – Grouped 50% | -0.673 | 0.030 | 23976 | -22.416 | < 0.001 |
|  | Ripple – Random – 50% | -1.175 | 0.030 | 23976 | -39.110 | < 0.001 |
|  | 5 cm high – Random – 50% | -0.113 | 0.030 | 23976 | -3.775 | 0.031 |
|  | 2.5 cm high – Random – 50% | -0.898 | 0.030 | 23976 | -29.874 | < 0.001 |
|  | 3 panel – Random – 50% | -0.851 | 0.030 | 23976 | -28.320 | < 0.001 |
| **2.5 cm high – Grouped – 50%** | Control | 0.674 | 0.029 | 6993 | 22.878 | < 0.001 |
|  | 3 panel – Grouped 50% | 0.192 | 0.030 | 23976 | 6.395 | < 0.001 |
|  | Ripple – Random – 50% | -0.309 | 0.030 | 23976 | -10.299 | < 0.001 |
|  | 5 cm high – Random – 50% | 0.752 | 0.030 | 23976 | 25.036 | < 0.001 |
|  | 2.5 cm high – Random – 50% | -0.032 | 0.030 | 23976 | -1.063 | 1 |
|  | 3 panel – Random – 50% | 0.015 | 0.030 | 23976 | 0.491 | 1 |
| **3 panel – Grouped – 50%** | Control | 0.482 | 0.030 | 6993 | 15.966 | < 0.001 |
|  | Ripple – Random – 50% | -0.502 | 0.030 | 23976 | -16.694 | < 0.001 |
|  | 5 cm high – Random – 50% | 0.560 | 0.030 | 23976 | 18.641 | < 0.001 |
|  | 2.5 cm high – Random – 50% | -0.224 | 0.030 | 23976 | -7.457 | < 0.001 |
|  | 3 panel – Random – 50% | -0.177 | 0.030 | 23976 | -5.904 | < 0.001 |
| **Ripple – Random – 50%** | Control | 0.983 | 0.031 | 6993 | 32.097 | < 0.001 |
|  | 5 cm high – Random – 50% | 1.062 | 0.030 | 23976 | 35.335 | < 0.001 |
|  | 2.5 cm high – Random – 50% | 0.277 | 0.030 | 23976 | 9.236 | < 0.001 |
|  | 3 panel – Random – 50% | 0.324 | 0.030 | 23976 | 10.790 | < 0.001 |
| **5 cm high – Random – 50%** | Control | -0.078 | 0.028 | 6993 | -2.745 | 0.006 |
|  | 2.5 cm high – Random – 50% | -0.784 | 0.030 | 23976 | -26.099 | < 0.001 |
|  | 3 panel – Random – 50% | -0.737 | 0.030 | 23976 | -24.545 | < 0.001 |
| **2.5 cm high – Random – 50%** | Control | 0.706 | 0.029 | 6993 | 23.962 | < 0.001 |
|  | 3 panel – Random – 50% | 0.047 | 0.030 | 23976 | 1.554 | 0.998 |
| **3 panel – Random – 50%** | Control | 0.659 | 0.030 | 6993 | 21.843 | < 0.001 |
